# Field and laboratory evaluation of Abbott-Bioline™ Malaria Ag *Pf/Pv* RDT performance in a high-transmission setting: contrasting results with a low-endemic area

**DOI:** 10.1186/s12936-026-05869-1

**Published:** 2026-03-18

**Authors:** Jocia Fenomanana, Anthony Ravoavy, Malalanandrianina Rakotoarisoa, Lucien Platon, Alioune Wade, Buze Chala, Yssimini Nadège Guillène Tibiri, Allan Muller, Lucas Thiebaut, Emmanuelle Caspar, Pierre-Emeric Strubel, Didier Ménard, Arsène Ratsimbasoa

**Affiliations:** 1 Faculty of Medicine, University of Fianarantsoa, Fianarantsoa, Madagascar; 2 Centre National d’Application de Recherches Pharmaceutiques, Antananarivo, Madagascar; 3 Malaria Genetics and Resistance Team (MEGATEAM), UR 3073 - Pathogens Host Arthropods Vectors Interactions Unit, University of Strasbourg, Strasbourg, France; 4 Biomedical and Public Health Department, Institut de Recherche en Sciences de La Santé, (IRSS), Ouagadougou, Burkina Faso; 5 Laboratoire de Biochimie Et Immunologie Appliquées (LABIA), Université Joseph KI- Zerbo, Ouagadougou, Burkina Faso; 6 Malaria Parasite Biology and Vaccines, INSERM Unit, 1347 - ParasitInnov, Institut Pasteur, Université Paris Cité, Paris, France; 7 Laboratory of Parasitology and Medical Mycology, CHU Strasbourg, Strasbourg, France; 8 Institut Universitaire de France (IUF), Paris, France

**Keywords:** Malaria, Rapid diagnostic test, HRP2, Diagnostic accuracy, Abbott-Bioline™ Malaria Ag *Pf/Pv*, Quality control, Madagascar

## Abstract

**Background:**

Recent reports documented catastrophic sensitivity failures (18%) of Abbott-Bioline™ Malaria Ag *Pf/Pv* rapid diagnostic tests (RDTs) at the Thailand-Myanmar border, prompting WHO to issue an information notice on quality concerns. We evaluated the same RDT lots under controlled laboratory conditions and in a high-transmission field setting in Madagascar to assess whether performance varied across epidemiological contexts.

**Methods:**

Laboratory evaluation tested four Abbott-Bioline™ Malaria Ag *Pf/Pv* lots (05DDI018BH, 05DDI020BA, 05DDI041AB, 05DDI040AA) using serial dilutions of cultured *Plasmodium falciparum* (0–60,784 parasites/µL). Field evaluation enrolled 218 consecutive febrile patients at Ranomafana health centre, southeastern Madagascar. Performance of both RDTs (Abbott-Bioline™ Malaria Ag *Pf/Pv* and Parascreen® Malaria Ag *Pf/Pan*) was assessed against microscopy and real-time PCR as reference standards.

**Results:**

In laboratory testing, substantial inter-lot variability was observed, with detection failures occurring between 97 and 373 parasites/µL. Incomplete blood migration and faint test lines were noted at lower densities. In the field, malaria prevalence was 70.2% by PCR and 48.6% by microscopy. Against microscopy, Abbott-Bioline™ Malaria Ag *Pf/Pv* achieved sensitivity of 99.1% (95% CI 94.9–100) and specificity of 93.7% (95% CI 87.4–97.4). Parascreen® Malaria Ag *Pf/Pan* showed sensitivity of 100% (95% CI 96.6–100) and specificity of 92.8% (95% CI 86.3–96.8). Against PCR, sensitivity decreased to 73.2% for Abbott-Bioline™ Malaria Ag *Pf/Pv* and 74.5% for Parascreen® Malaria Ag *Pf/Pan*, while specificity remained 98.5% for both tests. No significant difference was observed between RDTs (p > 0.05).

**Conclusions:**

Despite using identical lots that showed 18% sensitivity at the Thailand-Myanmar border, Abbott-Bioline™ Malaria Ag *Pf/Pv* RDTs achieved 99.1% sensitivity in Madagascar. This difference likely reflects higher parasite densities in the Malagasy high-transmission setting (geometric mean 10,006 parasites/µL) compared to low-transmission elimination contexts. Both RDTs met WHO performance thresholds against microscopy but missed approximately 25% of PCR-positive infections. These findings demonstrate that RDT performance is highly context-dependent and underscore the need for enhanced post-deployment surveillance.

**Supplementary Information:**

The online version contains supplementary material available at 10.1186/s12936-026-05869-1.

## Background

Malaria remains a major public health challenge globally, with an estimated 282 million cases and 610,000 deaths in 2024 [1]. In Madagascar, the malaria burden has escalated sharply, with an estimated 6.2 million cases (range: 4–8.7 million) and 16,000 deaths (range: 6,200–29,900) in 2024. Between 2023 and 2024, the country experienced a dramatic 27.7% increase in incidence, contributing an additional 1.9 million cases and ranking among the three countries (with Ethiopia and Yemen) accounting for 58% of the global case increase. Extreme climate events have disrupted health services and intervention delivery, further exacerbating this surge [1]. Of the 31.2 million population, 88% (27.4 million) live in high-transmission areas (> 1 case per 1,000 population), where *Plasmodium falciparum* dominates, transmitted by *Anopheles funestus* s.s., *An. gambiae* s.l., *An. arabiensis*, and *An. mascarensis* [2]. Transmission patterns vary spatially, from perennial transmission in coastal lowlands to epidemic-prone seasonal patterns in highland areas, highlighting the need for context-specific diagnostic tools.

Prompt and accurate diagnosis is essential for reducing malaria morbidity and mortality while preventing inappropriate antimalarial use. Since 2010, WHO has recommended parasitological confirmation of all suspected cases before treatment [3]. While microscopy remains the reference standard when performed by trained personnel, its capacity is limited in peripheral settings due to equipment and staffing constraints. Rapid diagnostic tests (RDTs) have thus substantially improved case management in remote areas due to their simplicity and rapid turnaround time [4, 5]. However, despite widespread deployment and WHO prequalification, recent field investigations have revealed alarming batch-specific performance failures, particularly for Abbott-Bioline™ Malaria Ag *Pf/Pv* RDTs in the Greater Mekong Subregion.

Aung et *al.* reported sensitivity of only 18% for *P. falciparum* and 44% for *P. vivax* at the Thailand-Myanmar border, with missed infections in febrile patients ranging from 512 to 75,360 parasites/µL [6]. These findings were independently confirmed by Thein et *al.* during a *P. vivax* outbreak in Northwest Myanmar, where the Abbott-Bioline™ Malaria Ag *Pf/Pv* RDT exhibited a similarly poor sensitivity of only 24% overall and 39% at parasite densities ≥ 200/µL, the WHO-recommended detection threshold [7]. This consistency across different epidemiological settings underscores a systemic issue with these RDT lots, which has received broad attention, including a Science news report detailing the accumulating evidence and institutional responses [8]. In response, the WHO Global Malaria Programme issued an internal memorandum (30 April 2025) identifying 11 affected lots, acknowledging that reports from ‘diverse field settings and experienced users’ indicated the issue was unrelated to user error [7, 8]. These findings expose critical gaps in RDT quality assurance, as prequalification testing of selected lots fails to guarantee sustained field performance across all manufacturing batches. This underscores the need for systematic post-market surveillance, combining laboratory assessment with field validation.

To address these critical quality concerns, we conducted a comprehensive evaluation of Abbott-Bioline™ Malaria Ag *Pf/Pv* RDT performance using the same lot numbers as those reported by Aung et *al.* [6], along with a comparative assessment against Parascreen® Malaria Ag *Pf/Pan* in Madagascar field conditions. The primary objective was to evaluate diagnostic performance of both RDTs against reference standards and determine whether identical Abbott-Bioline™ Malaria Ag *Pf/Pv* lots could maintain acceptable performance in different epidemiological contexts. Secondary objectives included characterizing inter-lot variability in laboratory conditions, a key factor potentially explaining the discrepancies observed in recent field studies [6, 7].

## Materials and methods

### Study design

This prospective diagnostic evaluation comprised two complementary phases: controlled laboratory assessment of Abbott-Bioline™ Malaria Ag *Pf/Pv* RDT lot-specific performance, and field validation comparing two RDT (Abbott-Bioline™ Malaria Ag *Pf/Pv* RDT and Parascreen® Malaria Ag *Pf/Pan*) against reference standards in a Madagascar clinical setting. The laboratory evaluation was conducted to assess inter-lot variability in detection thresholds, while the field study evaluated real-world diagnostic performance under operational conditions.

### Laboratory evaluation

#### Setting and materials

Laboratory evaluation was conducted at the University of Strasbourg, France. Four Abbott-Bioline™ Malaria Ag *Pf/Pv* lots (05DDI018BH, 05DDI020BA, 05DDI041AB, 05DDI040AA) were assessed using cultured *Plasmodium falciparum* parasites (strain 3601, collected in Pailin, Cambodia, 2010). These lots were identical to those reported by Aung et *al.* in their Thailand-Myanmar evaluation [6].

#### Serial dilution protocol

Parasite density was quantified by flow cytometry using SYBR Green I DNA staining [9]. Nine serial dilutions were prepared ranging from 0 to 60,784 parasites/µL (0, 19, 49, 97, 194, 373, 1,621, 5,708, and 60,784 parasites/µL). Five replicate RDTs were tested for each concentration per lot, totaling 180 tests allocated for laboratory evaluation.

#### Performance assessment

All tests were performed strictly following manufacturer instructions for use, including the use of 5 µL blood (50% hematocrit), addition of four drops of assay diluent, and result interpretation within the specified reading time (15–20 min) [10]. Test line intensity was scored using a standardized 5-point scale: 0 = no visible line (negative), 1 = very faint line (barely visible), 2 = faint line (clearly visible), 3 = moderate intensity (equal to control line), 4 = strong intensity (exceeding control line). Scoring was performed by two independent readers blinded to parasite concentration and lot identification. RDTs were stored at 4 °C according to manufacturer specifications, with continuous temperature monitoring.

### Field Evaluation

#### Setting and study population

**Fig. 1:**
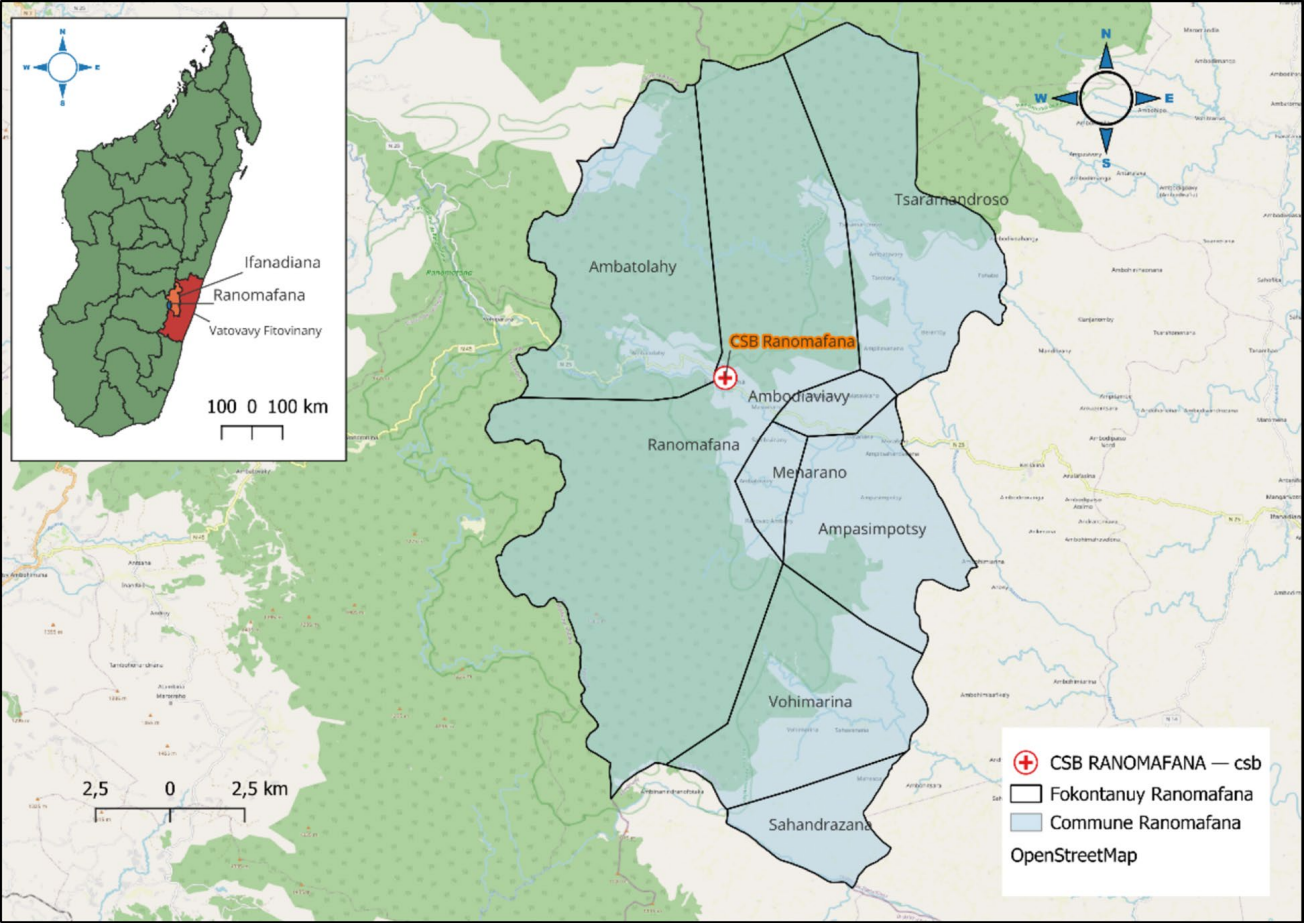
Geographic location of Ranomafana health center, Madagascar. Map showing the location of Ranomafana Health Center in southeastern Madagascar, where field evaluation of RDTs was conducted. Inset shows Madagascar's position relative to the African continent and Indian Ocean

Field evaluation was conducted at Ranomafana Health Center, southeastern Madagascar (21°15′S, 47°27′E, altitude 876 m), from February 20–28, 2025 (Fig. 1). This area represents stable, high-intensity malaria transmission with year-round *Plasmodium falciparum* predominance. Consecutive patients presenting with fever (≥ 38 °C) or fever history within 48 h were enrolled. Exclusion criteria included refusal to participate and previous enrollment during the study period.

#### Sample size and clinical procedures

A total of 218 patients were enrolled during the nine-day study period, determined by logistical constraints corresponding to available Abbott-Bioline™ Malaria Ag *Pf/Pv* RDT quantities. Capillary blood collection was performed for RDTs testing, microscopy slide preparation, and dried blood spot (DBS) collection for PCR analysis.

#### RDT testing procedures

*RDT specifications.* Two RDT were evaluated: Parascreen® Malaria Ag *Pf/Pan* (Zephyr Biomedicals, India; lots 101,612 and 101,675) used in routine at the health center and Abbott-Bioline™ Malaria Ag *Pf/Pv* (Abbott Diagnostics, Republic of Korea; lots 05DDI018BH, 05DDI020BA, 05DDI041AB, 05DDI040AA). Parascreen® Malaria Ag *Pf/Pan* detects HRP2 and pan-specific LDH antigens, while Abbott-Bioline™ Malaria Ag *Pf/Pv* detects HRP2 and *P. vivax*-specific LDH antigens.

*Testing Protocol*. All RDTs were performed according to manufacturer instructions by trained technicians blinded to other diagnostic results. Reading times were 20 min for Parascreen® Malaria Ag *Pf/Pan* and 15 min for Abbott-Bioline™ Malaria Ag *Pf/Pv*. RDTs were stored at 4 °C. All tests were used within expiration dates. Technical characteristics of both RDTs are summarized in *Supplementary Table S1.*

#### Reference methods

*Microscopy.* Thick and thin blood films were prepared and stained with 10% Giemsa solution. Microscopic examination was performed by two experienced microscopists blinded to RDTs and PCR results. Parasite density quantification followed WHO standard procedures: parasites/µL = (parasites counted × 8,000)/white blood cells counted for thick smears, and parasites/µL = (parasitized RBCs × 5,000,000)/(20 fields × 250 RBCs) for thin smears. Slides were declared negative after examination of 500 microscopic fields. Discordant results were resolved by a third senior microscopist.

All positive microscopy slides were reviewed by a WHO-certified microscopist from the National Malaria Control Program (NMCP) laboratory. Discordant results underwent examination by a second expert microscopist, with persistent discrepancies resolved by a third impartial reader.

*PCR analysis.* DNA extraction from DBS was performed using QIAamp 96 DNA Blood Kit (Hilden, Germany) following manufacturer protocols. Molecular detection employed a two-step real-time PCR approach targeting mitochondrial *cytochrome b* gene for *Plasmodium* genus screening, followed by species-specific PCR with external amplification for *P. falciparum*, *P. vivax*, *P. malariae*, and *P. ovale* identification [11]. Primers used for genus screening were RTPCRScreening2_F (5'-tggagtggatggtgttttaga-3') and RTPCRScreening2_R (5'-ttgcaccccaatarctcattt-3'). Nested real-time PCR used RTPCRScreening2_F and RTPCRSreening3_R (5'-accctaaaggatttgtgctacc-3'). Species-specific primers were: *P. falciparum*: Pf_RTPCR_F (5'-atggatatctggattgattttatttatga-3') and Pf_RTPCR_R (5'-tcctccacatatccaaattactgc-3'); *P. vivax*: Pv_RTPCR_F (5'-tgctacaggtgcatctcttgtattc-3') and Pv_RTPCR_R (5'-atttgtccccaaggtaaaacg-3'); *P. malariae*: Pm_RTPCR_F (5'-acaggtgcatcacttgtattttttc-3') and Pm_RTPCR_R (5'-tgctggaattgaagataataaattagtaataact-3'); *P. ovale*: Po_RTPCR_F (5'-gttatatggttatgtggaggatatactgtt-3') and Po_RTPCR_R (5'-cgaatggaagaataaaatgtagtacg-3'). Melt curve analysis confirmed amplification specificity. Quality controls were integrated throughout all analytical procedures, including negative controls at both extraction and PCR steps, and positive controls for each *Plasmodium* species sourced from an in-house biobank (WHO quality controls, UKNEQAS).

#### Statistical analysis

Data were analyzed using RStudio Desktop version 2025.05.0–496. Agreement between diagnostic methods was assessed using Cohen's kappa coefficient (κ). Sensitivity, specificity, positive predictive value (PPV), negative predictive value (NPV), and corresponding 95% confidence intervals were calculated using the Wilson score method. McNemar tests were used to compare paired differences between RDTs on the same samples. Chi-square tests compared RDT performance across patient subgroups. Inter-lot variability in laboratory evaluation was assessed using one-way ANOVA. Statistical significance was set at p ≤ 0.05.

***Ethical considerations***

Participation was voluntary with written informed consent obtained from participants or guardians for minors. Participant data confidentiality was maintained throughout. All malaria-positive cases received immediate treatment according to national guidelines (artesunate-amodiaquine). The study was approved by the National Malagasy Ethical committee (N.44 MSANP/SG/AMM/CERBM, March, 11, 2024).

## Results

### Laboratory evaluation of Abbott-Bioline™ Malaria Ag *Pf/Pv* RDT performance.

#### RDT lot characteristics

Four Abbott-Bioline™ Malaria Ag *Pf/Pv* RDT lots were evaluated: 05DDI018BH, 05DDI020BA, 05DDI041AB, and 05DDI040AA (Table 1). These lots are identical to those reported by Aung et *al.* [6]. A total of 180 RDTs were tested using *P. falciparum* serial dilutions (strain 3601), with 220 additional tests allocated for field evaluation in Madagascar.

**Table 1:** Abbott-Bioline™ Malaria Ag Pf/Pv RDT lot specifications

Lot Number	Manufacturing Date	Expiry Date	Quantity Tested	Storage Conditions	Temperature Range
05DDI018BH	25/05/2023	23/05/2025	100 tests	2–30 °C,	18–25 °C monitored
05DDI020BA	28/06/2023	23/05/2025	100 tests	2–30 °C,	18–25 °C monitored
05DDI041AB	19/02/2024	17/02/2026	100 tests	2–30 °C,	18–25 °C monitored
05DDI040AA	16/02/2024	14/02/2026	100 tests	2–30 °C,	18–25 °C monitored

#### Detection threshold performance by lot

**Fig. 2:**
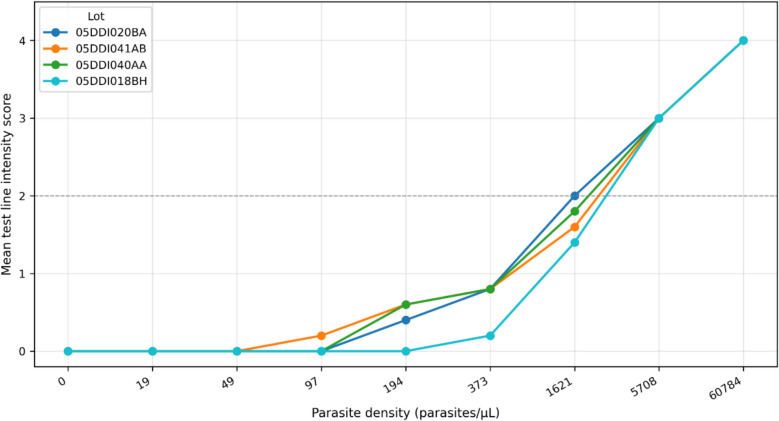
Detection threshold performance curves by Abbott-Bioline™ Malaria Ag Pf/Pv RDT Lot. Detection performance curves showing mean scoring intensity versus parasite density for four Abbott-Bioline™ Malaria Ag *Pf/Pv* lots. Each curve represents identical RDT brand and model but different manufacturing lots. Each data point represents the mean score of 5 replicate tests per concentration per lot. *X-axis: parasite density concentrations (0, 19, 49, 97, 194, 373, 1,621, 5,708, and 60,784 parasites/µL). Y-axis: Scoring scale: 0* = *No visible line (negative), 1* = *Very faint line (barely visible), 2* = *Faint line (clearly visible), 3* = *Moderate intensity (equal to control line), 4* = *Strong intensity (exceeding control line). Each curve represents identical RDT brand and model but different manufacturing lots*

Significant inter-lot variability was observed across the four Abbott-Bioline™ Malaria Ag *Pf/Pv* RDT lots. Line intensity decreased rapidly with declining parasite density across all lots. At 60,784 parasites/µL, all lots achieved maximum intensity (score 4). At 5,708 parasites/µL, all lots scored 3. At 1,621 parasites/µL, lot-specific variations emerged: lot 05DDI041AB showed 2/5 tests scoring 1, lot 05DDI040AA had 1/5 scoring 1, and lot 05DDI018BH had 3/5 scoring 1. At 373 parasites/µL, all lots showed at least one negative: 05DDI020BA (1/5), 05DDI041AB (1/5), 05DDI040AA (1/5), and 05DDI018BH (4/5). At 194 parasites/µL: 05DDI020BA 2/5 positive, 05DDI041AB 3/5 positive, 05DDI040AA 3/5 positive, 05DDI018BH 0/4 positive. At 97 parasites/µL, only lot 05DDI041AB produced one positive result (1/5) (Table 2*, *Fig. 2*)*.

**Table 2:** Abbott-Bioline™ Malaria Ag Pf/Pv RDT Performance by Lot and Parasite Density

Lot	Diluant	Parasites/µL	RDT1	RDT2	RDT3	RDT4	RDT5
05DDI020BA	05BDDI065	60,784	**4**	**4**	**4**	**4**	**4**
05DDI020BA	05BDDI065	5708	3	3	3	3	3
05DDI020BA	05BDDI065	1621	2	2	2	2	2
05DDI020BA	05BDDI065	373	1	1	1	1	**0**
05DDI020BA	05BDDI065	194	1	**0**	**0**	**0**	1
05DDI020BA	05BDDI065	97	**0**	**0**	**0**	**0**	**0**
05DDI020BA	05BDDI065	49	**0**	**0**	**0**	**0**	**0**
05DDI020BA	05BDDI065	19	**0**	**0**	**0**	**0**	**0**
05DDI020BA	05BDDI065	0	**0**	**0**	**0**	**0**	**0**
05DDI041AB	05BDDJ013	60,784	**4**	**4**	**4**	**4**	**4**
05DDI041AB	05BDDJ013	5708	3	3	3	3	3
05DDI041AB	05BDDJ013	1621	2	2	1	1	2
05DDI041AB	05BDDJ013	373	1	1	1	1	**0**
05DDI041AB	05BDDJ013	194	1	1	**0**	1	**0**
05DDI041AB	05BDDJ013	97	**0**	**0**	**0**	1	**0**
05DDI041AB	05BDDJ013	49	**0**	**0**	**0**	**0**	**0**
05DDI041AB	05BDDJ013	19	**0**	**0**	**0**	**0**	**0**
05DDI041AB	05BDDJ013	0	**0**	**0**	**0**	**0**	**0**
05DDI040AA	05BDDJ001	60,784	**4**	**4**	**4**	**4**	**4**
05DDI040AA	05BDDJ001	5708	3	3	3	3	3
05DDI040AA	05BDDJ001	1621	2	2	2	2	1
05DDI040AA	05BDDJ001	373	1	1	1	1	**0**
05DDI040AA	05BDDJ001	194	1	1	**0**	**0**	1
05DDI040AA	05BDDJ001	97	**0**	**0**	**0**	**0**	**0**
05DDI040AA	05BDDJ001	49	**0**	**0**	**0**	**0**	**0**
05DDI040AA	05BDDJ001	19	**0**	**0**	**0**	**0**	**0**
05DDI040AA	05BDDJ001	0	**0**	**0**	**0**	**0**	**0**
05DDI018BH	05BDDI070	60,784	**4**	**4**	**4**	**4**	**4**
05DDI018BH	05BDDI070	5708	3	3	3	3	3
05DDI018BH	05BDDI070	1621	2	2	1	1	1
05DDI018BH	05BDDI070	373	**0**	**0**	1	**0**	**0**
05DDI018BH	05BDDI070	194	**0**	**0**	**0**	**0**	**x**
05DDI018BH	05BDDI070	97	**0**	**0**	**0**	**0**	**0**
05DDI018BH	05BDDI070	49	**0**	**0**	**0**	**0**	**0**
05DDI018BH	05BDDI070	19	**0**	**0**	**0**	**0**	**0**
05DDI018BH	05BDDI070	0	**0**	**0**	**0**	**0**	**0**

Individual RDT performance across four Abbott-Bioline™ Malaria Ag *Pf/Pv* lots at different parasite concentrations

*Scoring system: 4* = *Strong intensity line (exceeding control line), 3* = *Moderate intensity line (equal to control line), 2* = *Faint line (*clearly* visible), 1* = *Very faint line (barely visible), 0* = *No visible line (negative), x* = *Invalid result (migration failure or technical issue)*

Migration defects were frequently observed, with incomplete blood migration leaving red background that cleared after 30 min in most cases (***Supplementary materials***).

### Field validation. Clinical performance at health center in Madagascar

#### Study population characteristics

A total of 218 patients presenting with suspected malaria were enrolled consecutively during the nine-day study period. The median age was 28 years (IQR: 15–45), with pediatric patients (< 15 years) comprising 50.4% (110/218) of the study population. Gender distribution was approximately equal (52.3% female, 47.7% male).

Fever was the predominant symptom, documented in 89.4% (195/218) of patients at enrollment, with 76.1% reporting fever onset within 48 h of presentation. The symptom profile included: headache (84.9%), chills (72.5%), body aches (68.8%), and nausea/vomiting (45.4%) were commonly reported. Treatment history revealed that 15.6% (34/218) of patients had taken antimalarial medication within the previous 30 days, predominantly artemether-lumefantrine. No patients reported severe malaria symptoms.

Parasite densities at enrollment were high overall, with a geometric mean of 10,006 parasites/µL (95% CI: 7,167–13,970), a median of 14,698 parasites/µL, and a wide range from 166 to 348,800 parasites/µL (Table 3).

**Table 3:** ***Patient Demographics and Clinical Characteristics****.* Baseline characteristics of 218 consecutive patients with suspected malaria enrolled at Ranomafana Health Center, Madagascar, during February 20–28, 2025

Variables	Number (%)
**Gender**	
Male	117 (53.7)
Female	101 (46.3)
**Age group (years)**	
< 5	40 (18.3)
5—15	70 (32.1)
≥ 15	108 (49.6)
**Clinical presentation**	
Fever documented	195 (89.4)
Fever onset < 48 h	166 (76.1)
Headache	185 (84.9)
Chills	158 (72.5)
Body aches	150 (68.8)
Nausea/vomiting	99 (45.4)
**Treatment history**	
Recent antimalarials (< 30 days)	34 (15.6)
**Parasite density (/µL)**	
Geometric mean (95%IC)	10,006 (7,167–13,970)
Range	166–348,800
Median	14,698

#### Diagnostic performance against reference standards (microscopy and PCR)

Among the 218 samples tested, prevalence varied by diagnostic method. PCR detected the highest proportion of infections at 70.2% (153/218), followed by Parascreen® Malaria Ag *Pf/Pan* at 52.8% (115/218), Abbott-Bioline™ Malaria Ag *Pf/Pv* at 51.8% (113/218), and microscopy at 48.6% (106/218). A total of 106 patients (48.6%) tested positive across all four diagnostic methods.

Species distribution analysis revealed *P. falciparum* predominance across all methods (98%, 150/153 *P. falciparum* and 2.0%, 3/150 *P. vivax*). PCR and microscopy detected three cases of *P. vivax* infections, all of which were also identified by Parascreen® Malaria Ag *Pf/Pan*, while Abbott-Bioline™ Malaria Ag *Pf/Pv* detected only two of these *P. vivax* cases. No discordant cases in species identification were observed between RDTs, microscopy, and PCR.

*Performance against microscopy.* Concordance analysis showed 96.3% agreement between both RDTs and microscopy. Parascreen® Malaria Ag *Pf/Pan* achieved sensitivity of 100% (95% CI: 96.6–100) with specificity of 92.8% (95% CI: 86.3–96.8), recording 8 false positives and no false negatives (κ = 0.93). Abbott-Bioline™ Malaria Ag *Pf/Pv* demonstrated sensitivity of 99.1% (95% CI: 94.9–100) and specificity of 93.7% (95% CI: 87.4–97.4), with 7 false positives and 1 false negative (κ = 0.93). The single missed case by Abbott-Bioline™ Malaria Ag *Pf/Pv* had parasitemia of 1,847 parasites/µL in the 1,001–5,000 parasites/µL density range. Statistical comparison revealed no significant difference between the two RDTs for any performance metric (p > 0.05) (Table 4).

**Table 4:** Concordance between RDT results and microscopy reference standard

	Microscopic examination		
Parascreen® Malaria Ag *Pf/Pan*	Positive n (%)	Negative n (%)	Total
Positive	107 (49.1)	8 (3.7)	115
Negative	0 (0)	103 (47.2)	103
Total	107	111	218
Abbott-Bioline™ Malaria Ag *Pf/Pv*	Positive n (%)	Negative n (%)	Total
Positive	106 (48.6)	7 (3.2)	113
Negative	1 (0.5)	104 (47.7)	105
Total	107	111	218

Cross-tabulation of RDT results against microscopy findings for 218 patients with suspected malaria at Ranomafana Health Center, Madagascar (February 2025)

*Numbers represent *absolute* counts with percentages of total study population (n* = *218) in parentheses.* Parascreen® Malaria Ag *Pf/Pan κ* = *0.93,* Abbott-Bioline™ Malaria Ag *Pf/Pv κ* = *0.93*

*Performance against PCR.* Parascreen® Malaria Ag *Pf/Pan* showed 81.7% concordance.

(178/218, κ = 0.63) with PCR, achieving sensitivity of 74.5% (95% CI: 66.8–81.2) and specificity of 98.5% (95% CI: 91.7–100), with 1 false positive and 39 false negatives (all *P. falciparum*). Abbott-Bioline™ Malaria Ag *Pf/Pv* demonstrated 80.7% concordance (176/218, κ = 0.61) with PCR, with sensitivity of 73.2% (95% CI: 65.5–80) and specificity of 98.5% (95% CI: 91.7–100), recording 1 false positive and 41 false negatives (40 *P. falciparum*, 1 *P. vivax*). No statistically significant differences were observed between RDTs (p > 0.05) (Table 5).

**Table 5:** Concordance between RDT results and PCR reference standard

	PCR		
Parascreen® Malaria Ag *Pf/Pan*	Positive n (%)	Negative n (%)	Total
Positive	114 (52.3)	1 (0.5)	115
Negative	39 (17.9)	64 (29.4)	103
Total	153	65	218
Abbott-Bioline™ Malaria Ag *Pf/Pv*	Positive n (%)	Negative n (%)	Total
Positive	112 (51.4)	1 (0.5)	113
Negative	41 (18.8)	64 (29.4)	105
Total	153	65	218

Cross-tabulation of RDT results against PCR findings for 218 patients with suspected malaria at Ranomafana Health Center, Madagascar (February 2025)

##### Numbers represent absolute counts with percentages of total study population (n = 218) in parentheses

*Parasite density stratification.* Against microscopy, Parascreen® Malaria Ag *Pf/Pan* maintained 100% sensitivity across all parasite density categories. Abbott-Bioline™ Malaria Ag *Pf/Pv* achieved 100% sensitivity in most categories except the 1,001–5,000 parasites/µL range (95.5% sensitivity). For submicroscopic infections (PCR-positive, microscopy-negative), both RDTs showed reduced sensitivity: 15.2% for Parascreen® Malaria Ag *Pf/Pan* and 13.0% for Abbott-Bioline™ Malaria Ag *Pf/Pv*.

*Predictive values.* Against microscopy, positive predictive values were 93% for Parascreen® Malaria Ag *Pf/Pan* and 93.8% for Abbott-Bioline™ Malaria Ag *Pf/Pv*, with negative predictive values of 100% and 99% respectively. Against PCR, positive predictive values were 99.1% for both RDTs, while negative predictive values were 62.1% (Parascreen® Malaria Ag *Pf/Pan*) and 61% (Abbott-Bioline™ Malaria Ag *Pf/Pv*) (Table 6).

**Table 6:** Diagnostic performance of RDT against microscopy and PCR reference standards

RDT	Sensitivity [95% CI]	Specificity [95% CI]	PPV [95% CI]	NPV [95% CI]
**Versus Microscopy**				
Parascreen® Malaria Ag *Pf/Pan*	100% [96.6–100]	92.8% [86.3–96.8]	93% [86.8–96.9]	100% [96.5–100]
Abbott-Bioline™ Malaria Ag *Pf/Pv*	99.1% [94.9–100]	93.7% [87.4–97.4]	93.8% [87.7–97.4]	99% [94.8–100]
**Versus PCR**				
Parascreen® Malaria Ag *Pf/Pan*	74.5% [66.8–81.2]	98.5% [91.7–100]	99.1% [95.2–100]	62.1% [52–71.5]
Abbott-Bioline™ Malaria Ag *Pf/Pv*	73.2% [65.5–80]	98.5% [91.7–100]	99.1% [95.1–100]	61% [50.9–70.3]

Sensitivity, specificity, positive predictive value (PPV), and negative predictive value (NPV) of Parascreen® Malaria Ag *Pf/Pan* and Abbott-Bioline™ Malaria Ag *Pf/Pv* evaluated against microscopy and PCR reference standards (n = 218). Statistical comparisons between RDTs showed no significant differences for any performance metric (McNemar test, p > 0.05)

## Discussion

This study demonstrates that both Parascreen® Malaria Ag *Pf/Pan* and Abbott-Bioline™ Malaria Ag *Pf/Pv* RDTs exhibited excellent diagnostic performance in a high-transmission setting in Madagascar. The evaluation compared these RDTs to microscopy and PCR in febrile patients from a stable transmission area characterized by a semi-immune population presenting with variable parasitemia levels (range from 166 to 348,800 parasites/µL). With sensitivities of 100% for Parascreen® Malaria Ag *Pf/Pan* and 99.1% for Abbott-Bioline™ Malaria Ag *Pf/Pv* against microscopy, both diagnostic tools substantially exceeded WHO-recommended thresholds (≥ 95% sensitivity and specificity) [12]. These performances align with many African studies, including in Madagascar, that consistently report high sensitivities for HRP2-based RDTs in stable endemic areas [13–19]. The current findings fall within the upper range of these reported values, confirming the reliability of both RDTs in similar epidemiological contexts.

The slightly reduced specificity (92.8% and 93.7% for Parascreen® Malaria Ag *Pf/Pan* and Abbott-Bioline™ Malaria Ag *Pf/Pv* respectively) resulted from false positive results (8 for Parascreen® Malaria Ag *Pf/Pan* and 7 for Abbott-Bioline™ Malaria Ag *Pf/Pv*) among microscopy-negative patients. These false positives likely reflect HRP2 antigen persistence following treatment, a well-documented phenomenon [20–24]. Given that 15.6% of the study cohort had received anti-malarial treatment within the preceding 30 days, this factor potentially contributed to the observed false positives. Specificities vary according to epidemiological context, typically showing very high values in low-transmission areas (98.8–99.8%) but reduced values in high-transmission areas for HRP2 tests (79.7–80.7%) due to antigen persistence after recovery [25]. The moderate proportion of recently treated patients in this study may explain the relatively higher specificity observed for both tests compared to other high-transmission settings.

Statistical analysis revealed no significant differences between the performances of the two RDTs, suggesting that either option could be selected based on logistical and economic considerations. This performance equivalence has been observed in other African studies comparing these RDTs [13]. The single case missed by Abbott-Bioline™ Malaria Ag *Pf/Pv* (parasitemia of 1,847 parasites/µL) remains within the range of occasional detection failures reported for this parasite density range [26].

It should be noted that RDTs are not designed to achieve the same level of detection as PCR. They are intended to perform equivalently to expert microscopy, which remains the WHO-recommended reference for clinical diagnosis. The comparison with PCR was included to characterize the full spectrum of infections present in the study population, including submicroscopic parasitemia, and should be interpreted in this context. When PCR served as the reference standard, RDT sensitivity predictably decreased (74.5% for Parascreen® Malaria Ag *Pf/Pan* and 73.2% for Abbott-Bioline™ Malaria Ag *Pf/Pv*), while specificity remained high (98.5% for both tests). This discrepancy reveals the primary limitation of current RDTs: their inability to detect submicroscopic infections [27–31]. Previous studies have similarly observed lower and variable sensitivities (37–88%) when comparing RDTs to PCR, particularly in asymptomatic individuals with submicroscopic infections [32, 33]. The dramatic performance decline for low-density infections becomes particularly critical in malaria elimination contexts [34]. However, in high-transmission areas like Madagascar, this limitation has less impact on clinical management of symptomatic cases, where the primary diagnostic need focuses on detecting clinically relevant parasitemia [35].

An often overlooked aspect of WHO diagnostic guidance is that when malaria remains clinically suspected despite a negative RDT result and no alternative diagnosis can be identified, referral for confirmatory microscopy is explicitly recommended [36]. This recommendation is directly relevant to the context described in this manuscript and should be reinforced in settings where RDT performance may be suboptimal. The performance gap between RDTs and PCR observed in this study raises broader questions about the appropriate use of conventional RDTs in very low prevalence settings, particularly for screening asymptomatic individuals. In such contexts, where parasite densities are typically low and submicroscopic infections predominate, conventional RDTs have limited utility and their negative predictive value becomes unreliable. For malaria elimination programs, more sensitive diagnostic tools are clearly needed. The development of ultrasensitive RDTs, particularly for *P. vivax* where HRP2-based detection is not applicable and pLDH-based tests show reduced sensitivity at low densities, represents an unmet priority. Until such tools become available and affordable at scale, conventional RDTs should be complemented by microscopy or molecular methods in elimination settings.

This study has several limitations that should be considered when interpreting the results. The study population consisted predominantly of symptomatic patients (89.4% with documented fever), which may have biased sensitivity estimates upward as RDTs more reliably detect higher parasitemia cases. The single-site design in a high-transmission area limits generalizability to other epidemiological contexts. Evaluation of *P. vivax* performance was constrained by the low prevalence of this species in the sample. While storage conditions were controlled, potential variations in field handling that might affect RDT performance cannot be entirely excluded.

Laboratory evaluation identified concerning inter-lot variability for Abbott-Bioline™ Malaria Ag *Pf/Pv* RDTs (lots 05DDI018BH, 05DDI020BA, 05DDI041AB, and 05DDI040AA). Inter-lot differences became evident at parasitemia below 1,621 parasites/µL, with lot 05DDI018BH showing the lowest sensitivity (0/4 detection at 194 parasites/µL) and lot 05DDI041AB the highest (1/5 detection at 97 parasites/µL). These lots originated from the same 2024 production batch supplied to the Shoklo Malaria Research Unit (SMRU) that was independently evaluated by Aung et *al.* during their investigation of diagnostic failures at the Thai-Myanmar border [6]. Concurrently, Thein et *al.* evaluated a different lot (05DDI027A, expiry September 2025) during a *P. vivax* outbreak in northwest Myanmar (December 2024), reporting a sensitivity of only 24% among febrile patients (11/46 microscopy-confirmed cases) and 39% for parasitemia ≥ 200/µL [7]. The Madagascar cohort exhibited a geometric mean parasite density of 10,006 parasites/µL (range: 166–348,800) among microscopy-confirmed cases, with only one false-negative result at 1,847 parasites/µL. In contrast, Aung et *al.* conducted their evaluation in both symptomatic clinic attendees (geometric mean density: 2,498 parasites/µL) and asymptomatic survey participants (geometric mean density: 157 parasites/µL) [6]. Their findings demonstrated that these same RDT lots failed to detect 59 of 64 *P. falciparum* cases in asymptomatic surveys (density range: 2–12,416 parasites/µL) and 11 of 20 cases in febrile patients (density range: 512–42,320 parasites/µL), resulting in an overall sensitivity of only 18%.

All three studies documented consistent physical defects in test line appearance. The current laboratory evaluations confirmed faint/absent test lines and incomplete blood migration previously documented by Aung et *al.* during external quality control assessments [6]. Thein et *al.* used ImageJ analysis to objectively confirm that visually negative RDTs produced no detectable signal despite parasitemia ranging from 200–5,200/µL, well above the WHO-recommended detection threshold of 200 parasites/µL [7]. While storage conditions were controlled in all settings, Aung et *al.* noted striking inter-lot variability: 88% (52/59) of positive Abbott-Bioline™ Malaria Ag *Pf/Pv* RDT results originated from a single lot (05DDI040AA). Controlled testing ruled out storage conditions as the explanatory factor, pointing instead to manufacturing inconsistencies [6]. The fact that Thein et *al.* observed nearly identical performance issues with an entirely different lot (05DDI027A) against *P. vivax* underscores the systemic nature of the manufacturing problem across both *Plasmodium* species and production batches [7]. The marked difference in field performance between the different epidemiological contexts, despite using the same RDT product, underscores how operational factors and parasite density distributions influence diagnostic accuracy.

Beyond diagnostic failure rates, Thein et *al.* documented severe clinical consequences in remote Myanmar areas, including five pediatric deaths from *P. falciparum* malaria (2022–2023) following repeatedly negative RDT results, and one medical assistant who developed severe malaria after four consecutive negative tests [7]. These findings illustrate the life-threatening implications of low-sensitivity RDTs in settings where microscopy is unavailable and referral is prohibitively difficult.

These complementary findings demonstrate that Abbott-Bioline™ Malaria Ag *Pf/Pv* RDT performance is highly context-dependent, maintaining acceptable sensitivity (99.1%) in high-transmission settings like Madagascar but showing dramatically reduced performance (18%−24%) in low-transmission elimination contexts as reported by Aung et *al.* and Thein et *al.* [6, 7]. The substantial inter-lot variability observed across all three studies, combined with identical lots producing vastly different field outcomes, reveals critical gaps in current WHO pre-qualification systems that evaluate selected lots under controlled conditions but fail to capture real-world performance variations [37]. This evidence underscores the urgent need for improve post-deployment monitoring and context-specific diagnostic strategies, with supplementary confirmation by microscopy or PCR warranted in elimination settings where missed diagnoses could undermine malaria control progress.

## Supplementary Information

Additional file1

## Data Availability

Aggregated data supporting the main findings are provided in the article and supplementary materials. Individual participant data are not publicly available due to patient confidentiality and ethical committee restrictions but may be made available upon reasonable request with appropriate institutional approval.
