# Supplementary material for: Field and laboratory evaluation of Abbott-Bioline™ Malaria Ag Pf/Pv RDT performance in a high-transmission setting: contrasting results with a low-endemic area
Source: Malar J. 2026 Mar 18;25:176. doi: 10.1186/s12936-026-05869-1 (PMC13112640; doi:10.1186/s12936-026-05869-1)
Supplement: Supplementary file 1 — Additional file1 [file 12936_2026_5869_MOESM1_ESM.pdf]

Supplementary Materials

Field and laboratory evaluation of Abbott-Bioline™ Malaria Ag *Pf/Pv* RDT performance in a high-transmission setting: contrasting results with a low-endemic area

Table of contents

Supplementary Table S1. Technical characteristics of RDTs used 2

Supplementary Methods. Laboratory evaluation of Abbott-Bioline™ Malaria Ag Pf/Pv RDT performance 2

Materials and Methods 2

Experimental setup. 2

Figure S1. Laboratory setup for RDT evaluation. 2

Preparation of parasite dilutions. 3

RDT interpretation and scoring 3

Figure S2. Standardized scoring system for test line intensity. 3

Results 4

Representative RDT results by parasite density. 4

Figure S3. RDT results at 0 parasites/µL (negative control). 4

All lots are negative (0/5 positive for all lots). 4

Figure S4. RDT results at 19 parasites/µL. 6

Figure S5. RDT results at 49 parasites/µL 8

Figure S6. RDT results at 97 parasites/µL. 10

Figure S7. RDT results at 194 parasites/µL. 12

Figure S8. RDT results at 373 parasites/µL. 14

Figure S9. RDT results at 1,621 parasites/µL. 16

Figure S10. RDT results at 5,708 parasites/µL. 18

Figure S11. RDT results at 60,784 parasites/µL. 20

Migration defects. 22

Supplementary Table S1. Technical characteristics of RDTs used

Table S1. Technical characteristics of RDTs used

Characteristics	Parascreen® Malaria Ag *Pf/Pan*	Abbott-Bioline™ Malaria Ag *Pf/Pv*
**Country of origin**	India	Republic of Korea
**Manufacturer**	Zephyr Biomedicals	Abbott Diagnostics
**Batch numbers**	101612 (35 units)<br>101675 (183 units)	05DDIO18B (55 units)<br>05DDIO20B (54 units)<br>05DDIO40A (54 units)<br>05DDIO41A (55 units)
**Detected antigens**	HRP2<br>panLDH (pan-specific)	HRP2<br>pvLDH (*P. vivax*-specific)
**Reading time**	20 min	15 min

Supplementary Methods. Laboratory evaluation of Abbott-Bioline™ Malaria Ag Pf/Pv RDT performance

Materials and Methods

Experimental setup.

Four Abbott-Bioline™ Malaria Ag Pf/Pv RDT lots (05DDI020BA, 05DDI041AB, 05DDI040AA, 05DDI018BH) were evaluated using serial dilutions of cultured Plasmodium falciparum parasites. A total of 180 RDTs were tested (45 per lot: 9 concentrations × 5 replicates per concentration).

Figure S1. Laboratory setup for RDT evaluation.
(A) Four Abbott-Bioline™ Malaria Ag Pf/Pv lots with corresponding buffer solutions arranged by lot number. (B) Organization of RDTs for systematic testing across nine parasite density concentrations (rows 1-9).

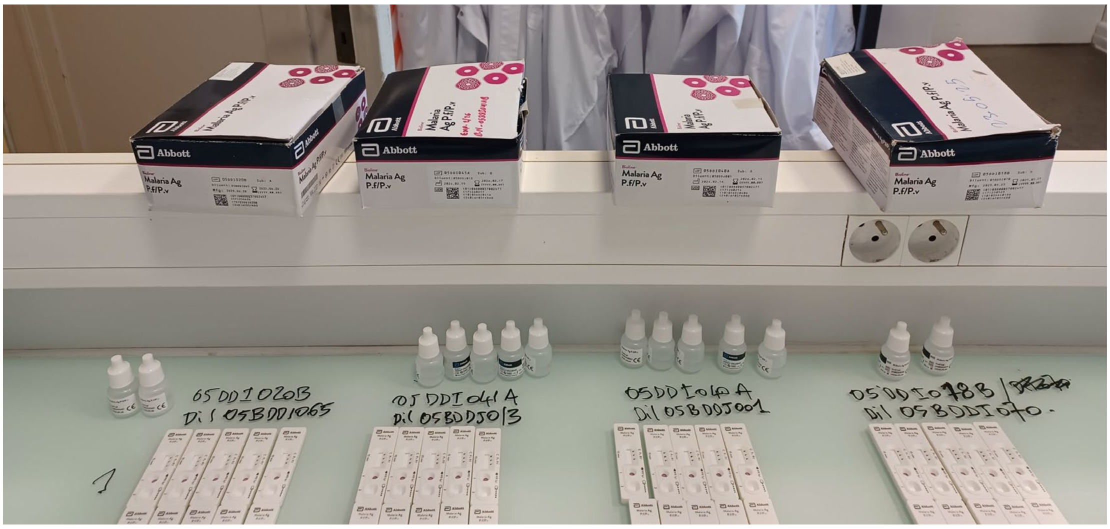

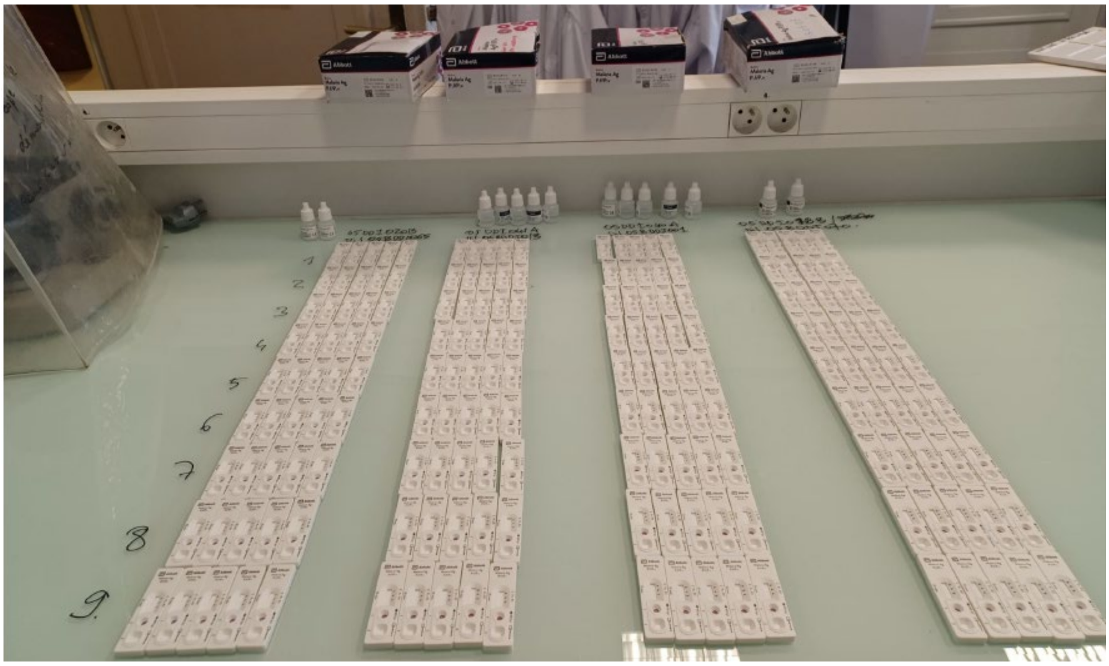

Preparation of parasite dilutions.

Parasite cultures were prepared using *P. falciparum* strain 3601 (collected in Pailin, Cambodia, 2010), a culture-adapted strain with confirmed absence of *pfhrp2/3* deletions. Parasites were synchronized at the 0-12h ring stage using sorbitol treatment. Serial dilutions were performed from stock cultures to achieve nine target concentrations: 0, 19, 49, 97, 194, 373, 1,621, 5,708, and 60,784 parasites/µL. Final parasite densities were quantified by flow cytometry using SYBR Green I DNA staining.

RDT interpretation and scoring

Figure S2. Standardized scoring system for test line intensity.
Results were scored on a 5-point scale: 4 = strong intensity (Pf line exceeding control line), 3 = moderate intensity (Pf line equal to control line), 2 = faint line (clearly visible), 1 = very faint line (barely visible), 0 = no visible Pf line (negative). Two independent readers scored each RDT, blinded to parasite concentration and lot identification.

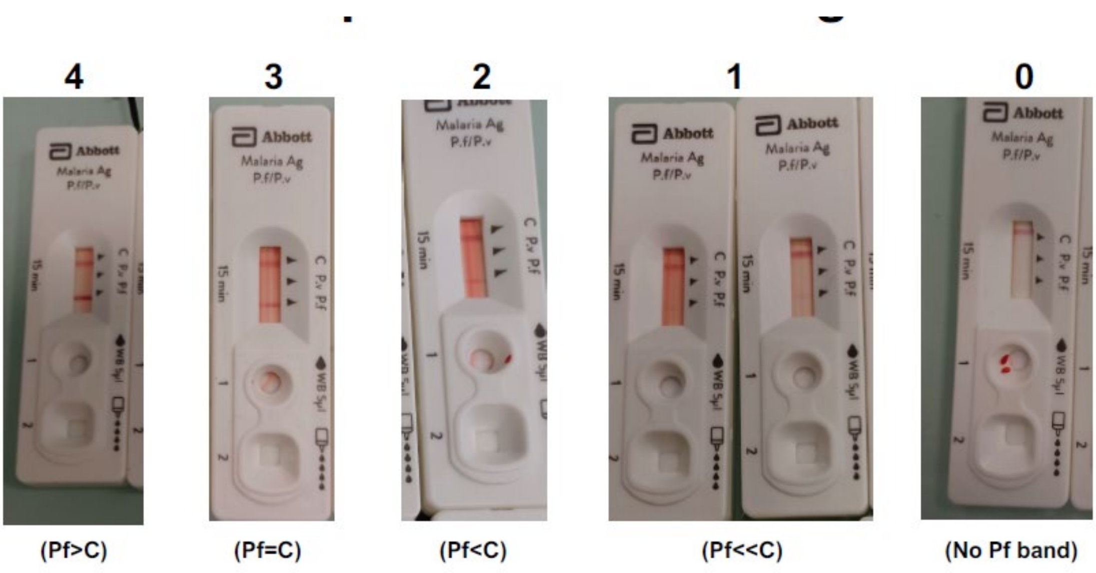

Results

Representative RDT results by parasite density.

The following figures show representative RDT results for each lot at different parasite densities, demonstrating inter-lot variability and detection thresholds.

Figure S3. RDT results at 0 parasites/μL (negative control).
All lots are negative (0/5 positive for all lots).

Lot 05DDI020BA (0/5 positive)

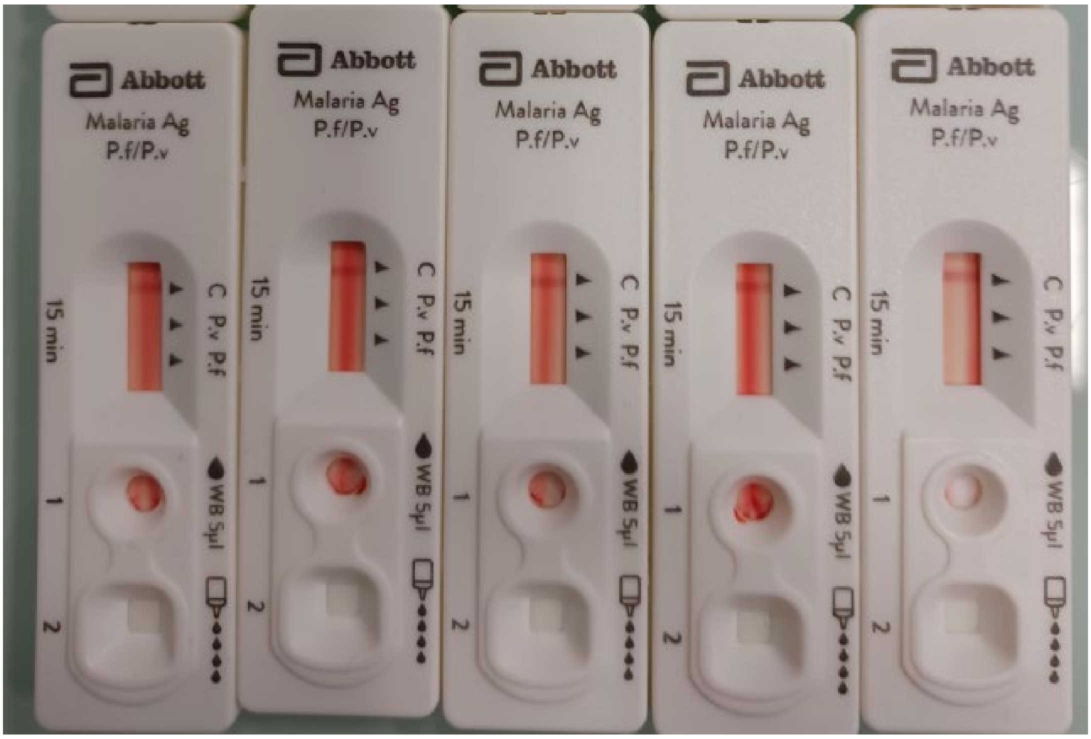

Lot 05DDI041AB (0/5 positive)

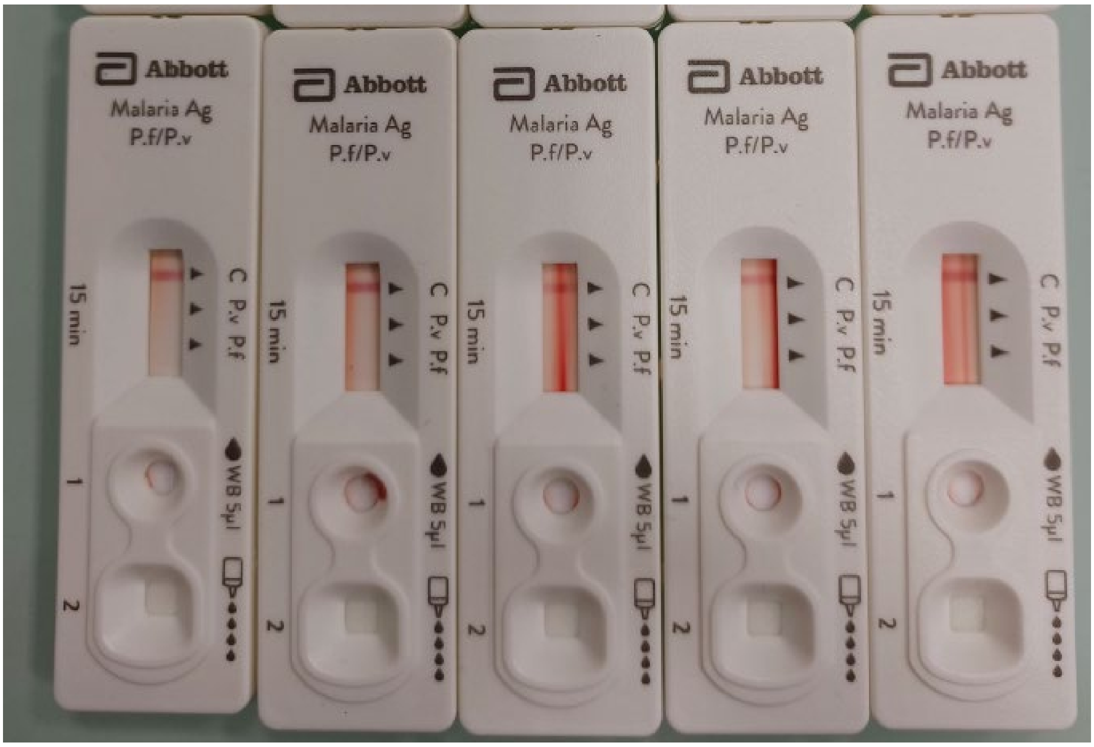

Lot 05DDI040AA (0/5 positive)

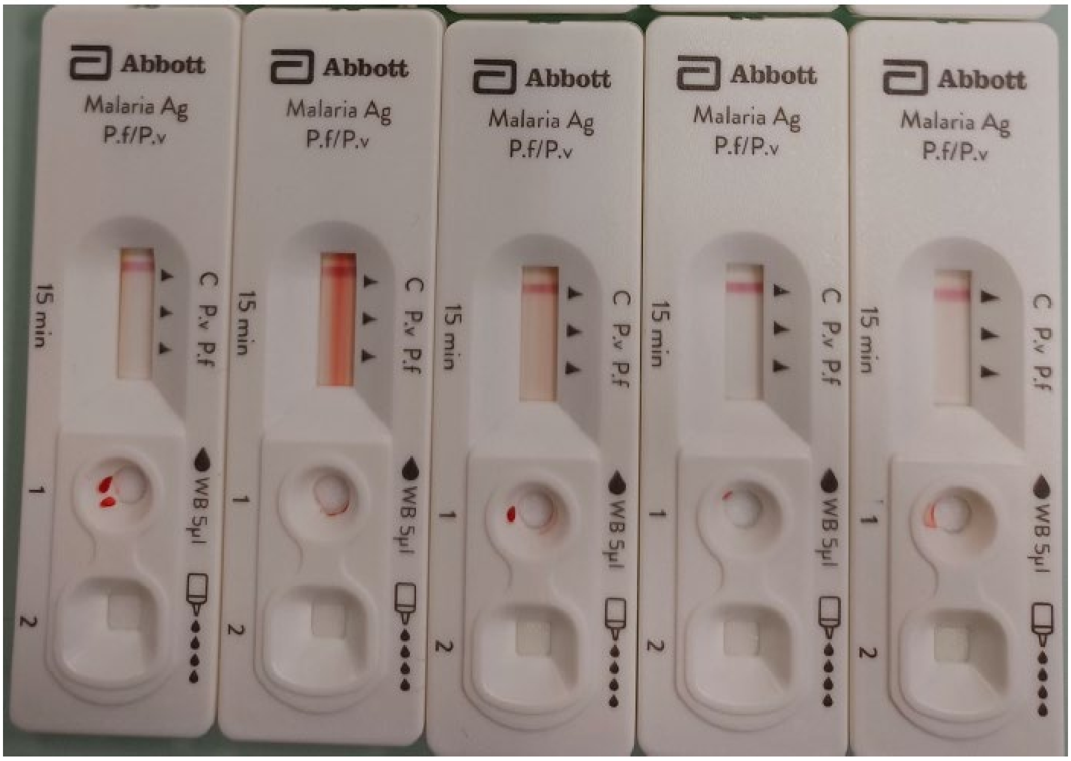

Lot 05DDI018BH (0/5 positive)

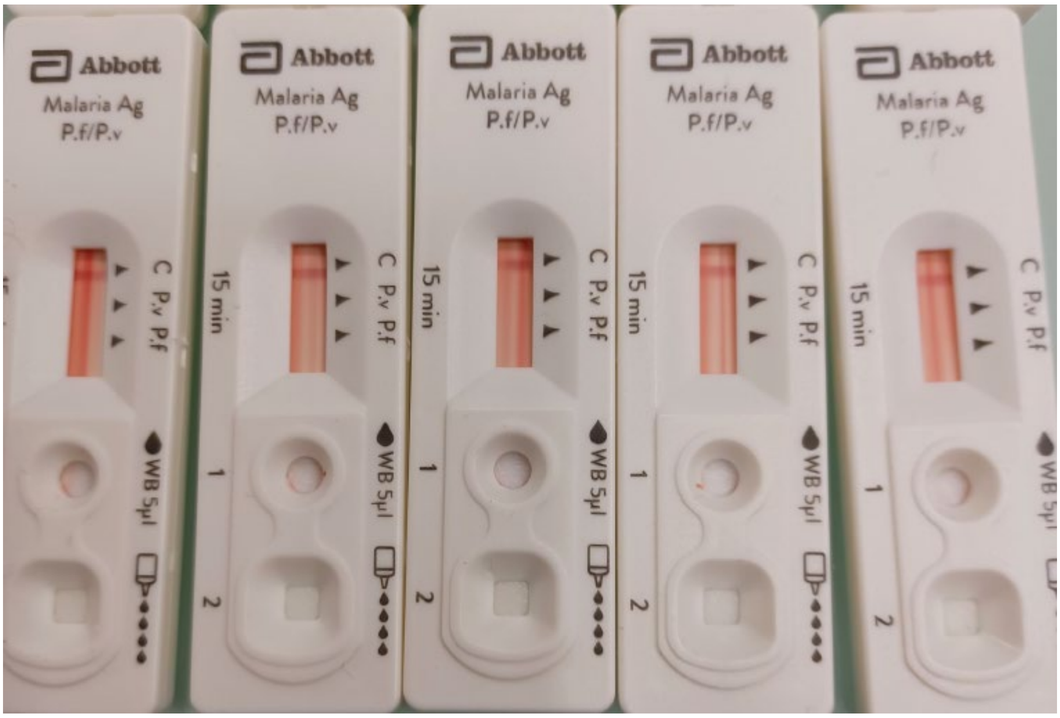

Figure S4. RDT results at 19 parasites/µL.
All lots are negative (0/5 positive for all lots).

Lot 05DDI020BA (0/5 positive)

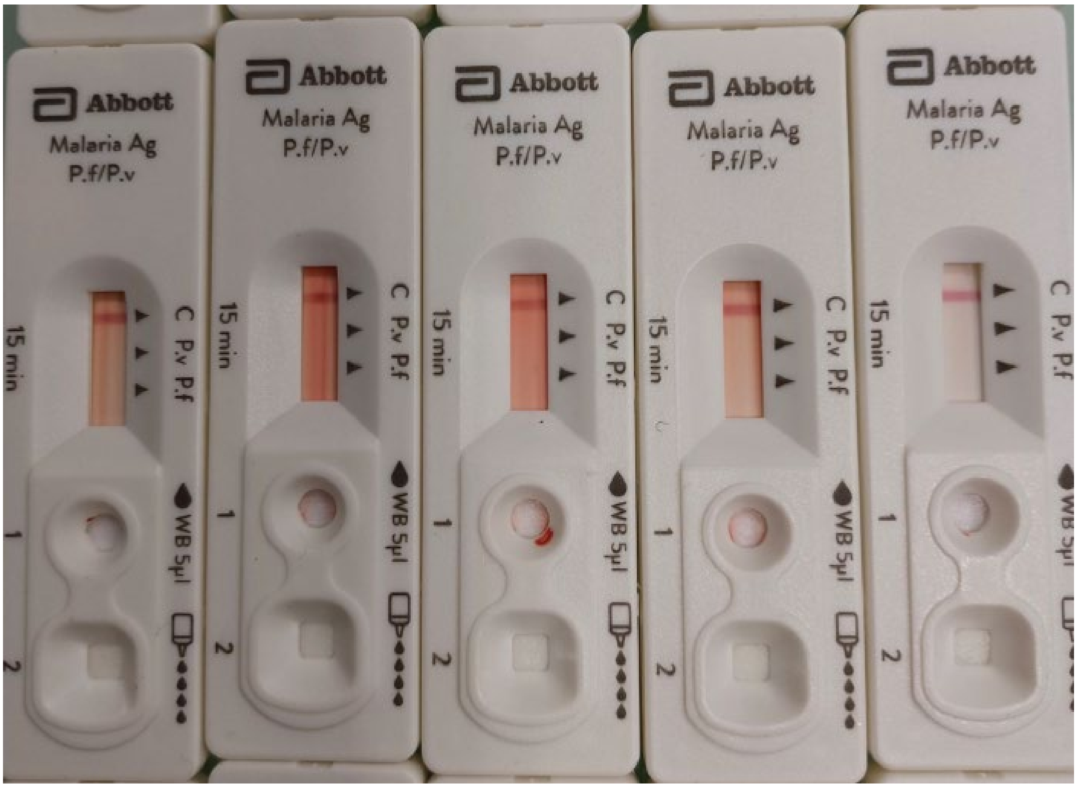

Lot 05DDI041AB (0/5 positive)

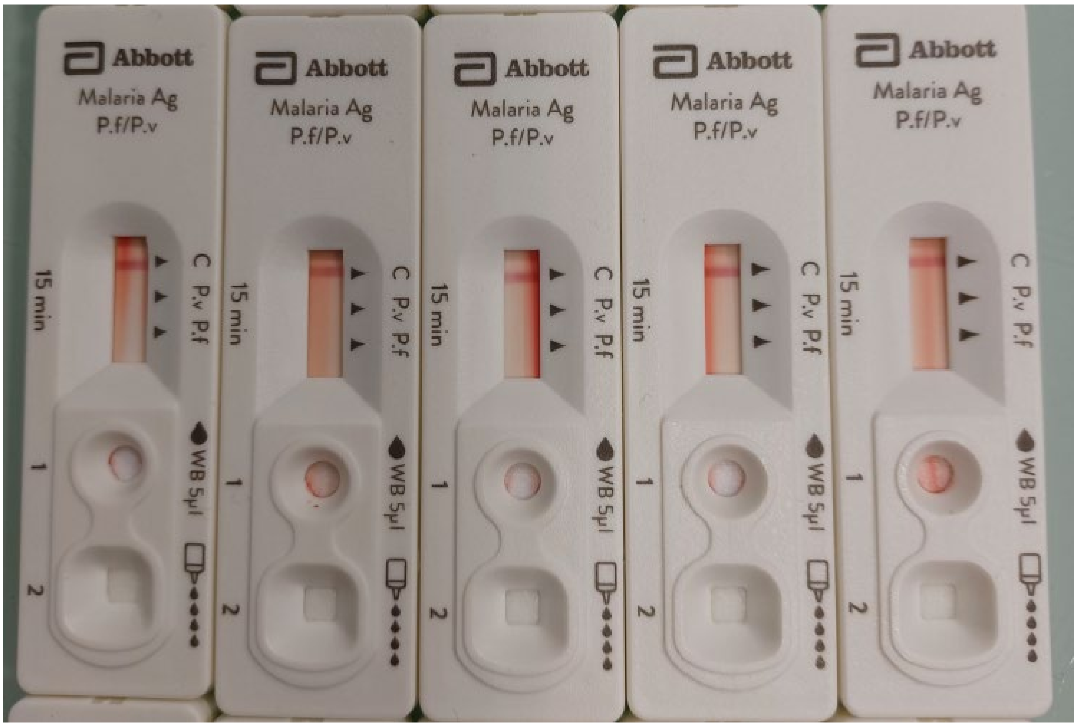

Lot 05DDI040AA (0/5 positive)

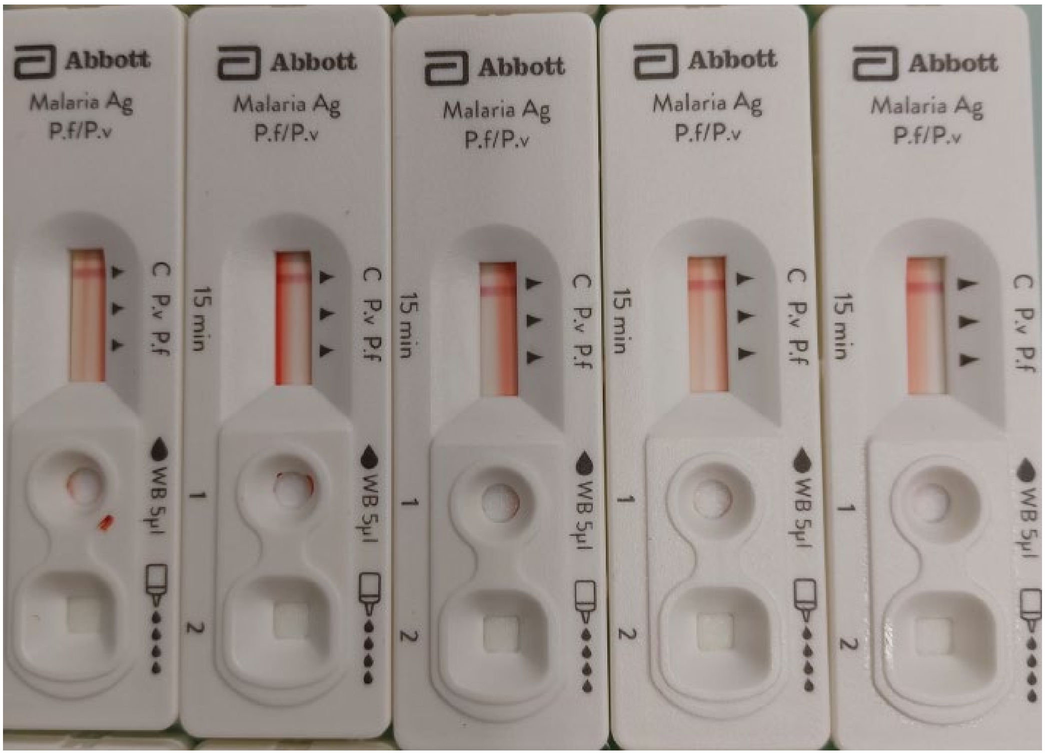

Lot 05DDI018BH (0/5 positive)

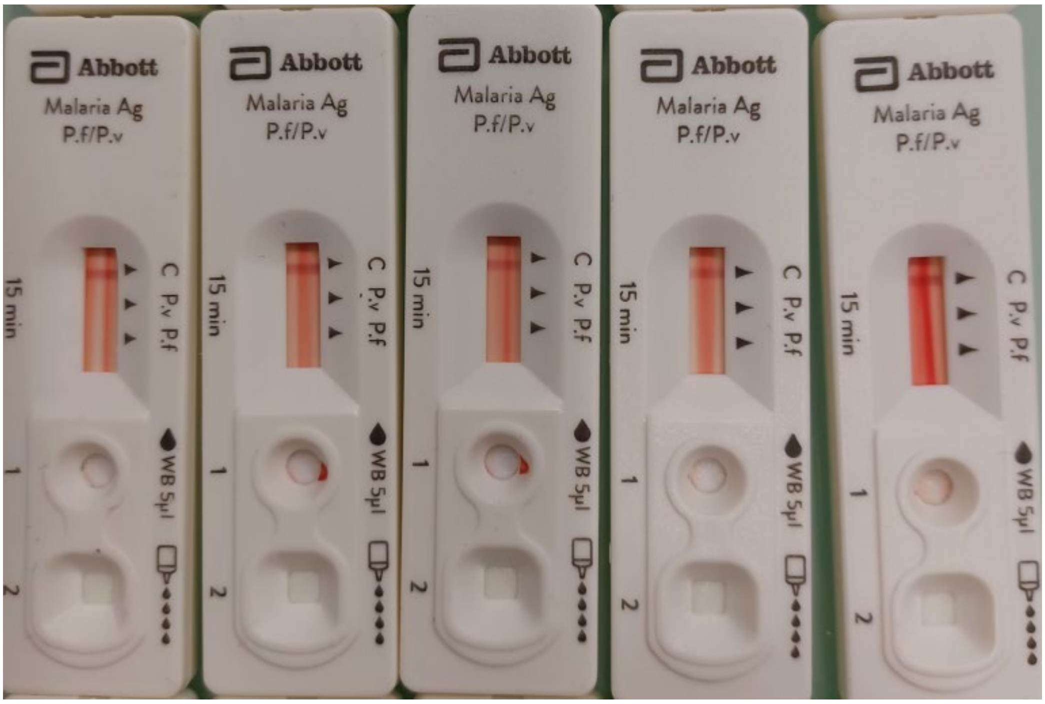

Figure S5. RDT results at 49 parasites/µL
All lots are negative (0/5 positive for all lots).

Lot 05DDI020BA (0/5 positive)

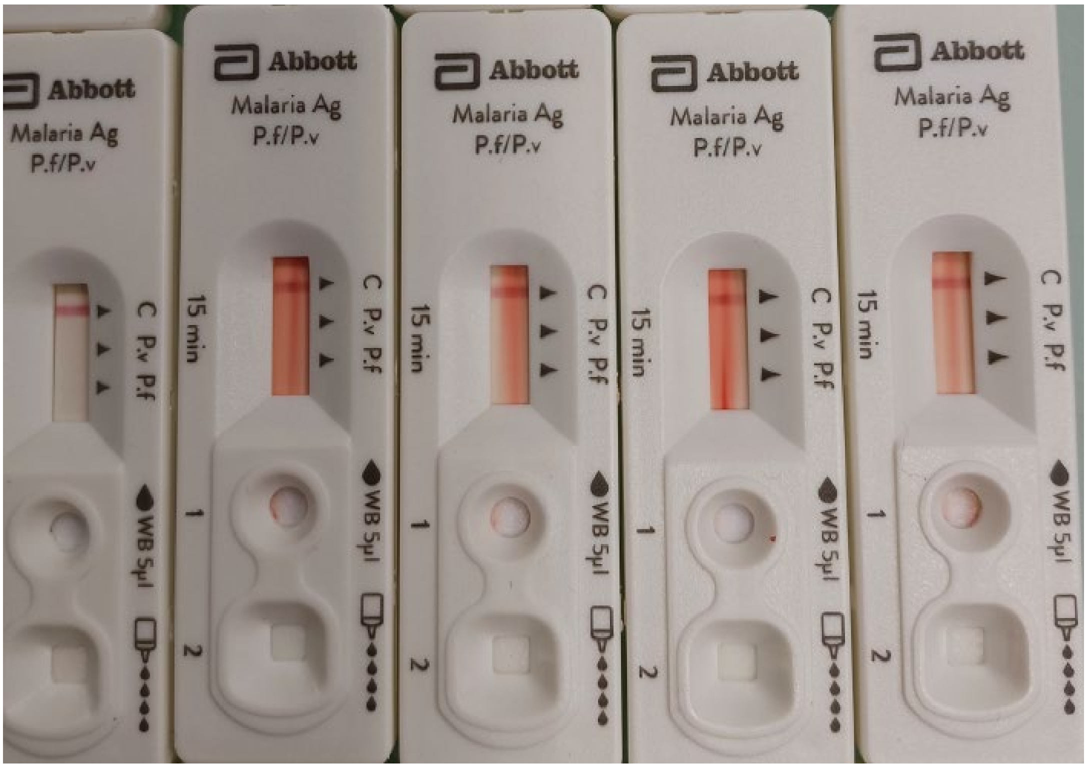

Lot 05DDI041AB (0/5 positive)

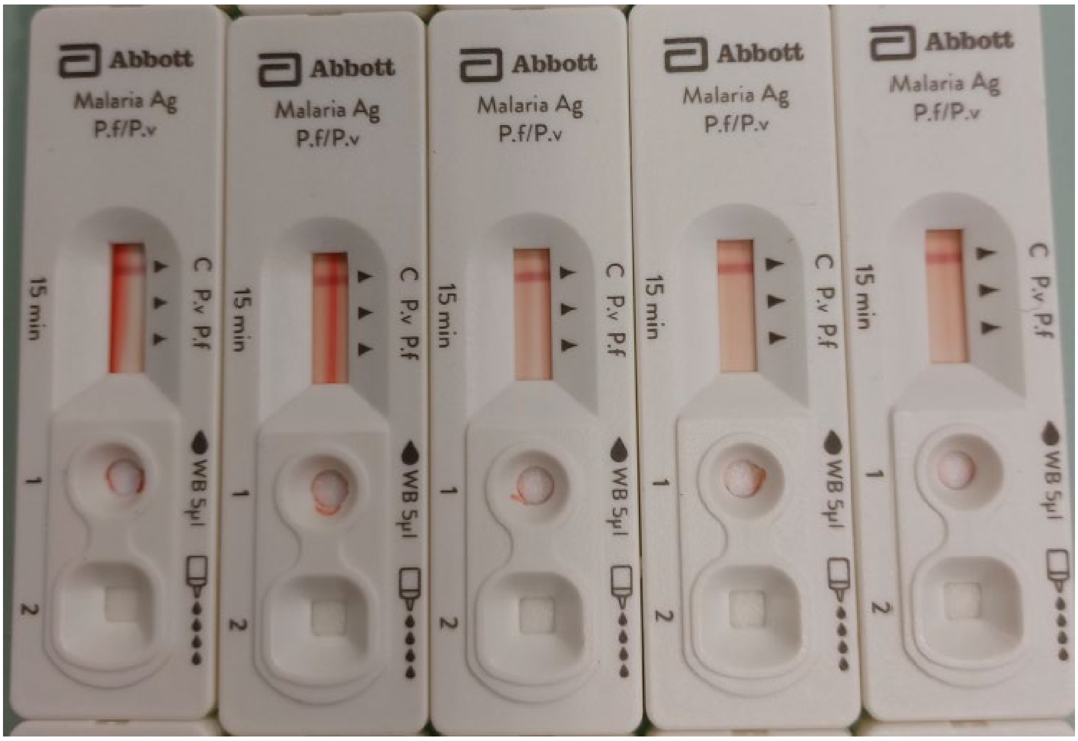

Lot 05DDI040AA (0/5 positive)

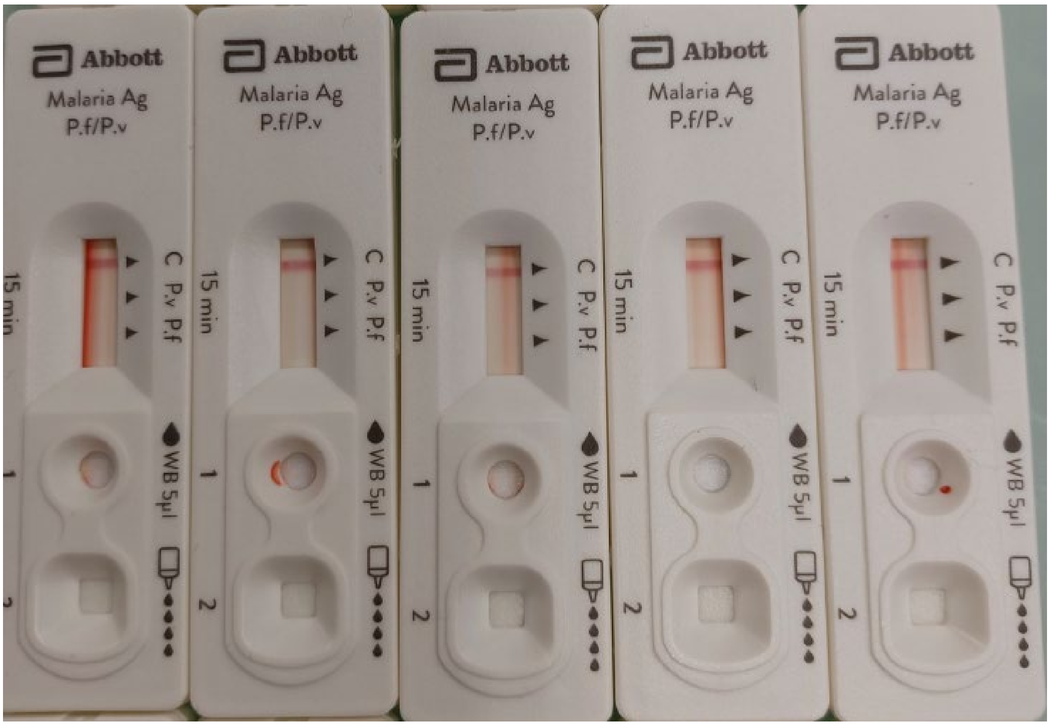

Lot 05DDI018BH (0/5 positive)

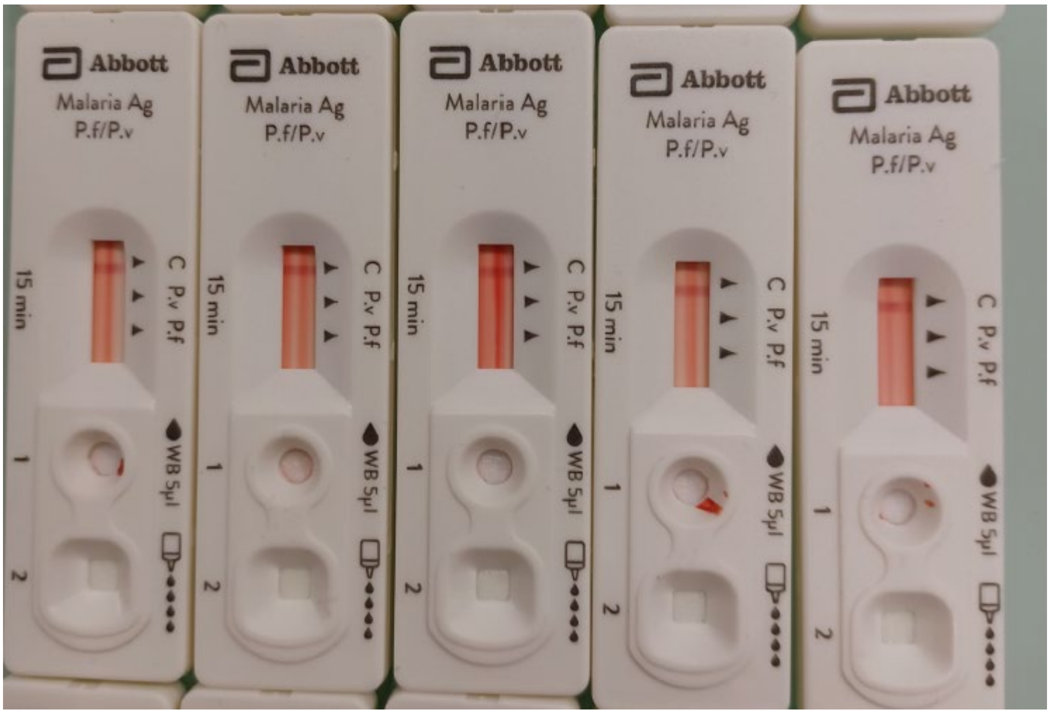

Figure S6. RDT results at 97 parasites/µL.
Emergence of inter-lot variability (lot 05DDI041AB: 1/5 positive; all other lots: 0/5 positive).

Lot 05DDI020BA (0/5 positive)

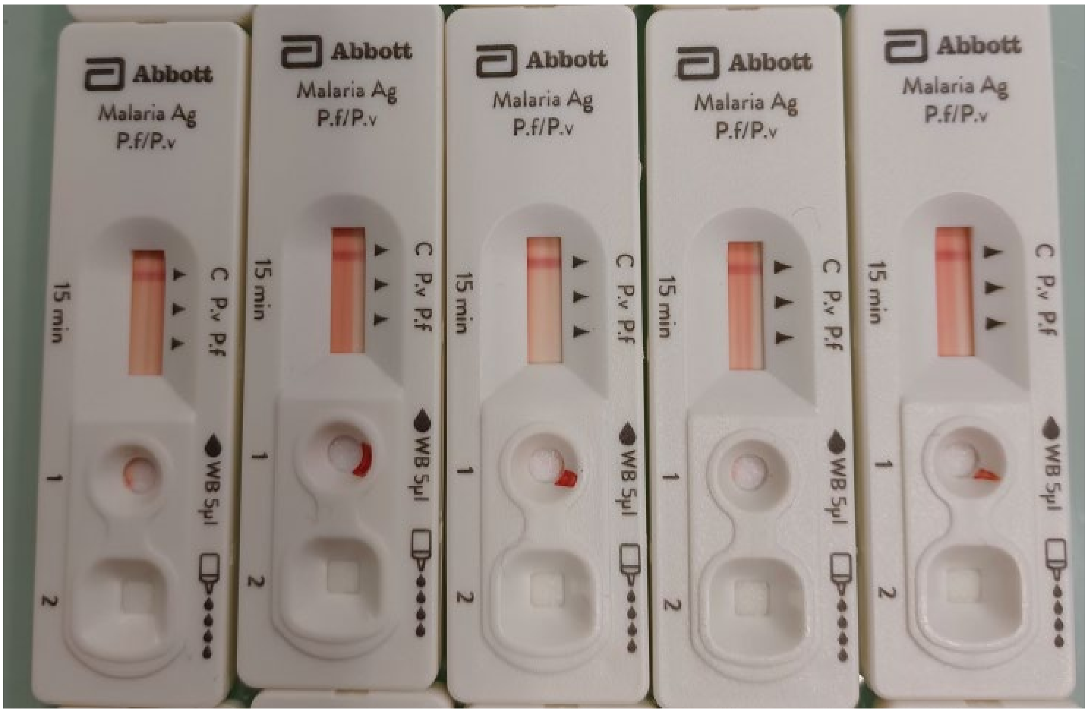

Lot 05DDI041AB (1/5 positive)

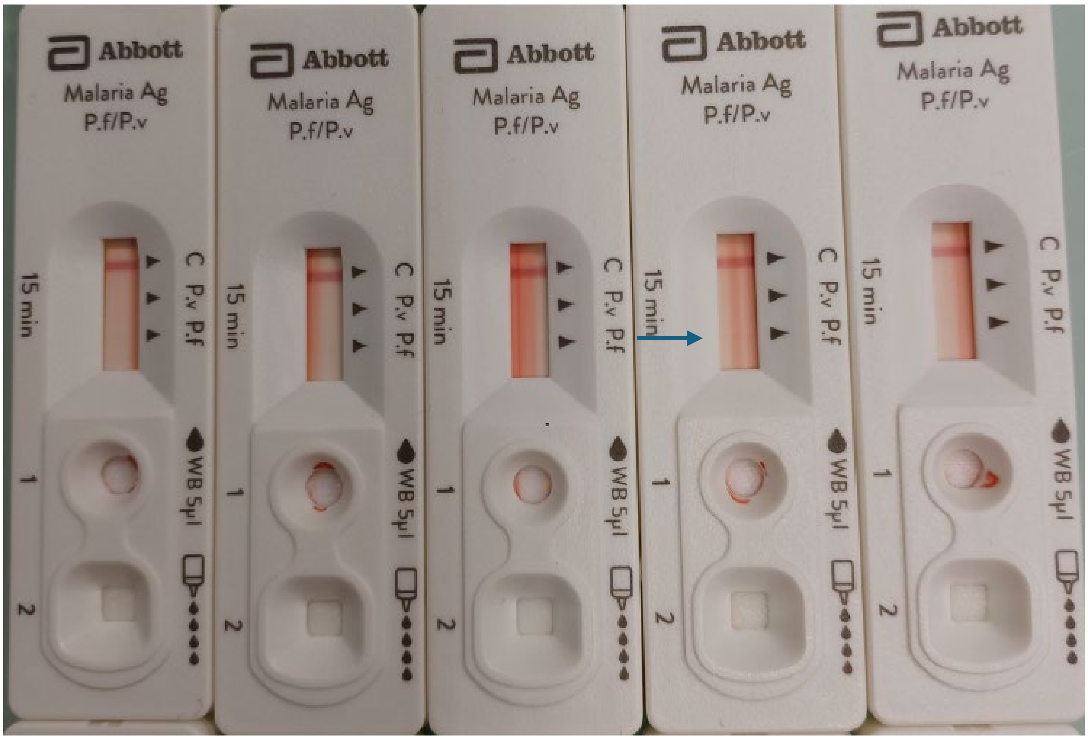

Lot 05DDI040AA (0/5 positive)

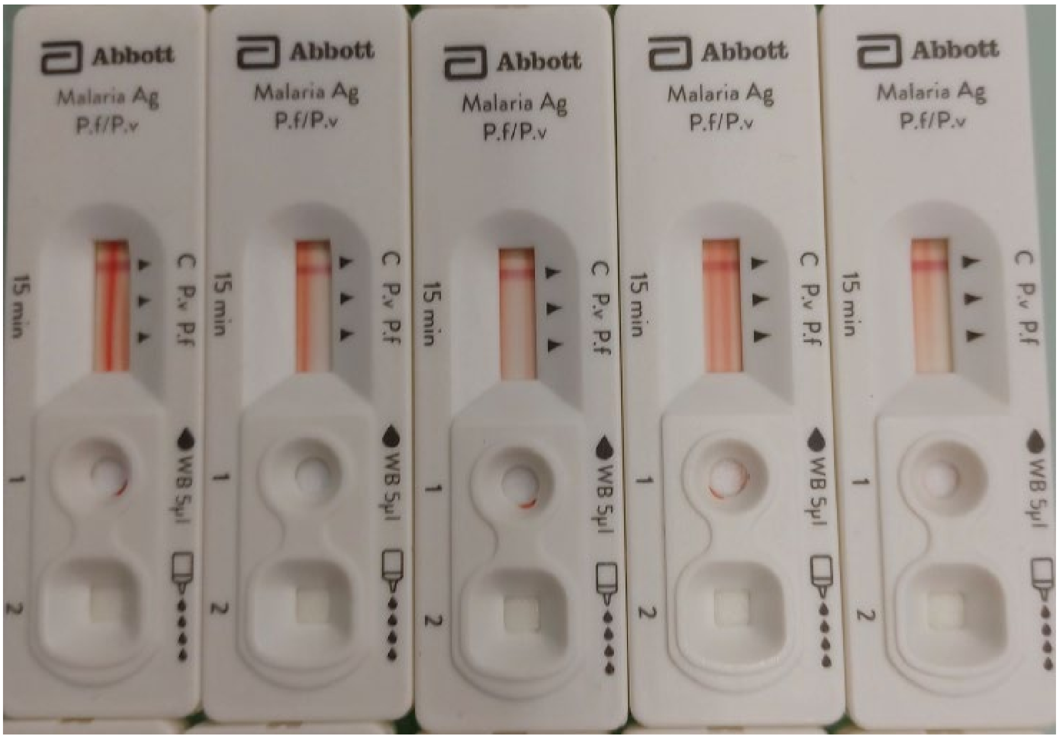

Lot 05DDI018BH (0/5 positive)

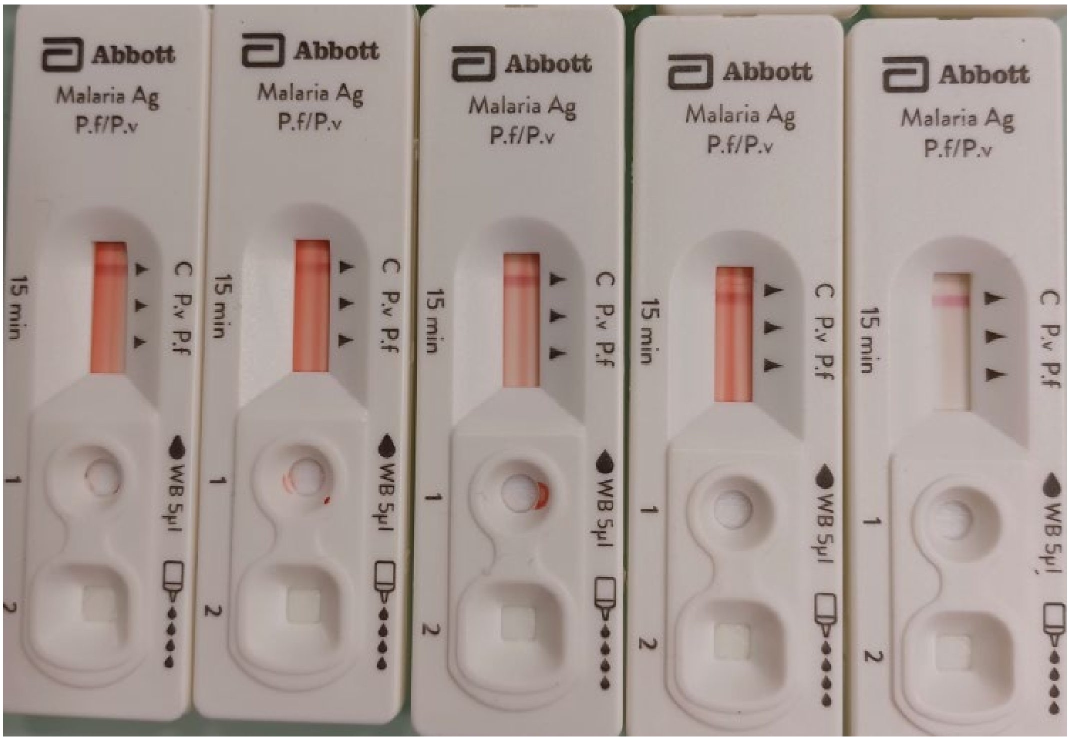

Figure S7. RDT results at 194 parasites/µL.
Increased inter-lot differences.

Lot 05DDI020BA (2/5 positive)

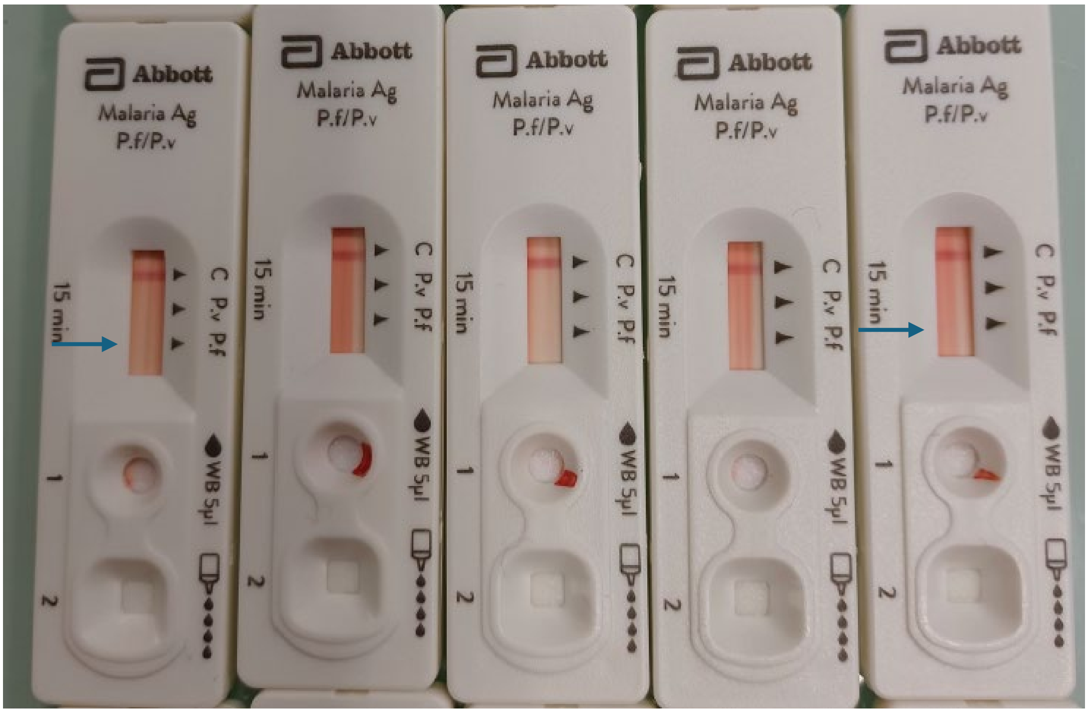

Lot 05DDI041AB (3/5 positive)

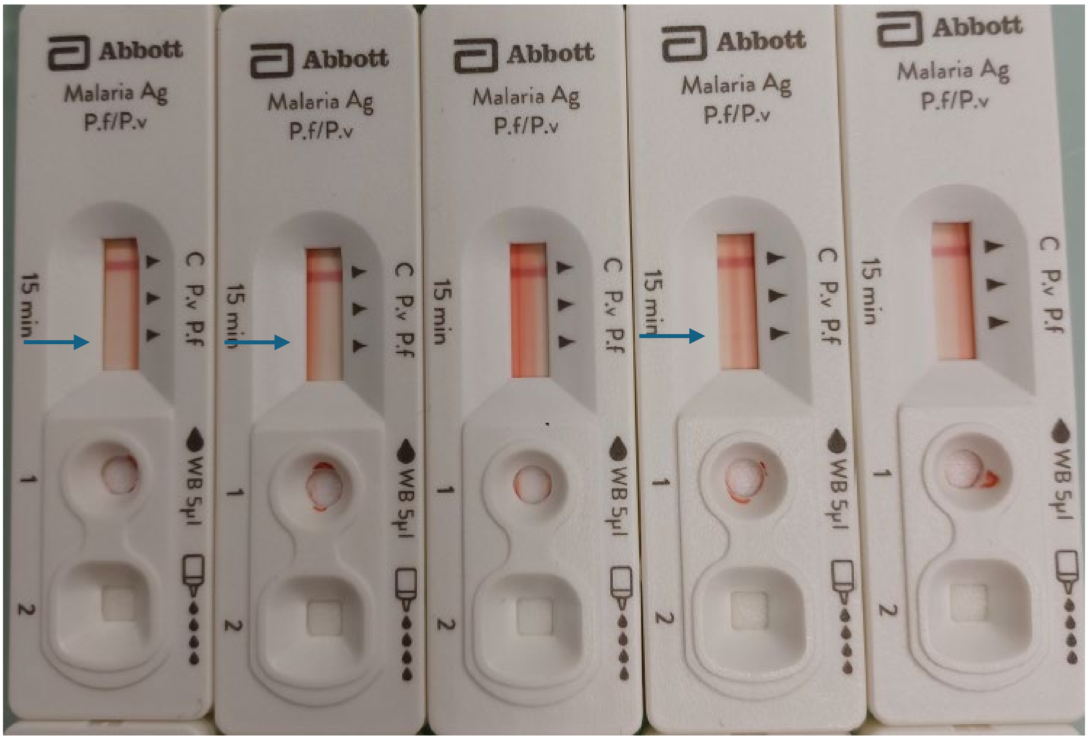

Lot 05DDI040AA (3/5 positive)

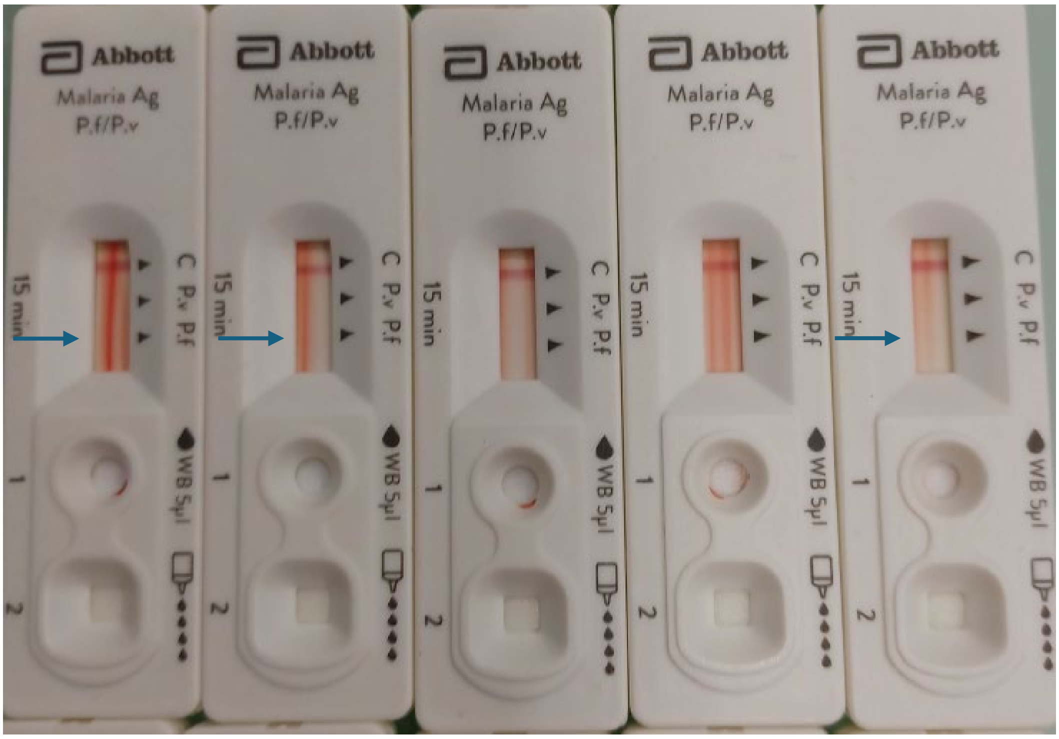

Lot 05DDI018BH (0/4 positive)

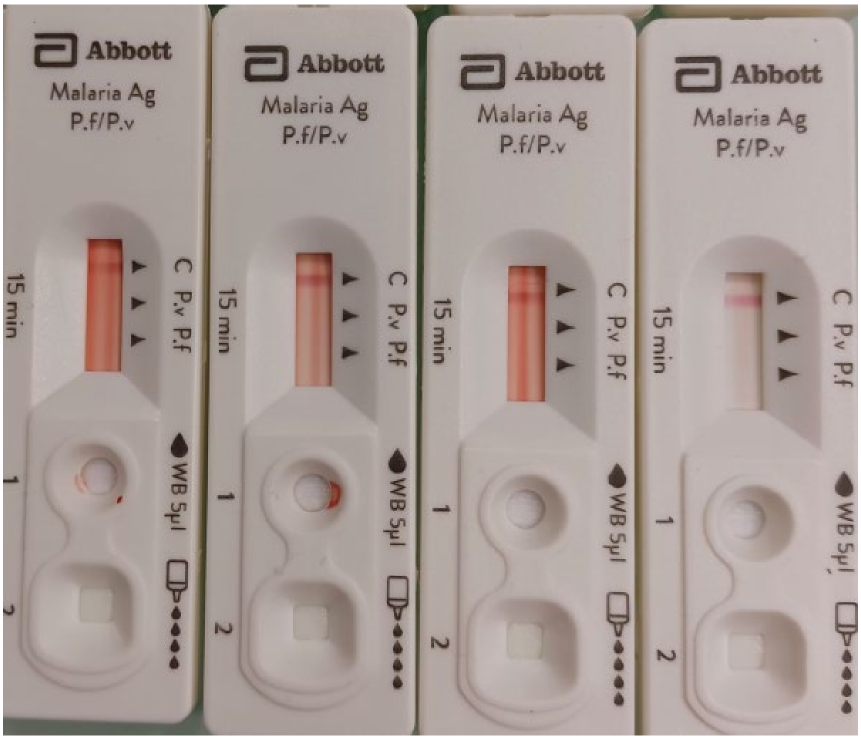

Figure S8. RDT results at 373 parasites/µL.
Consistent detection challenges at WHO-recommended threshold.

Lot 05DDI020BA (4/5 positive)

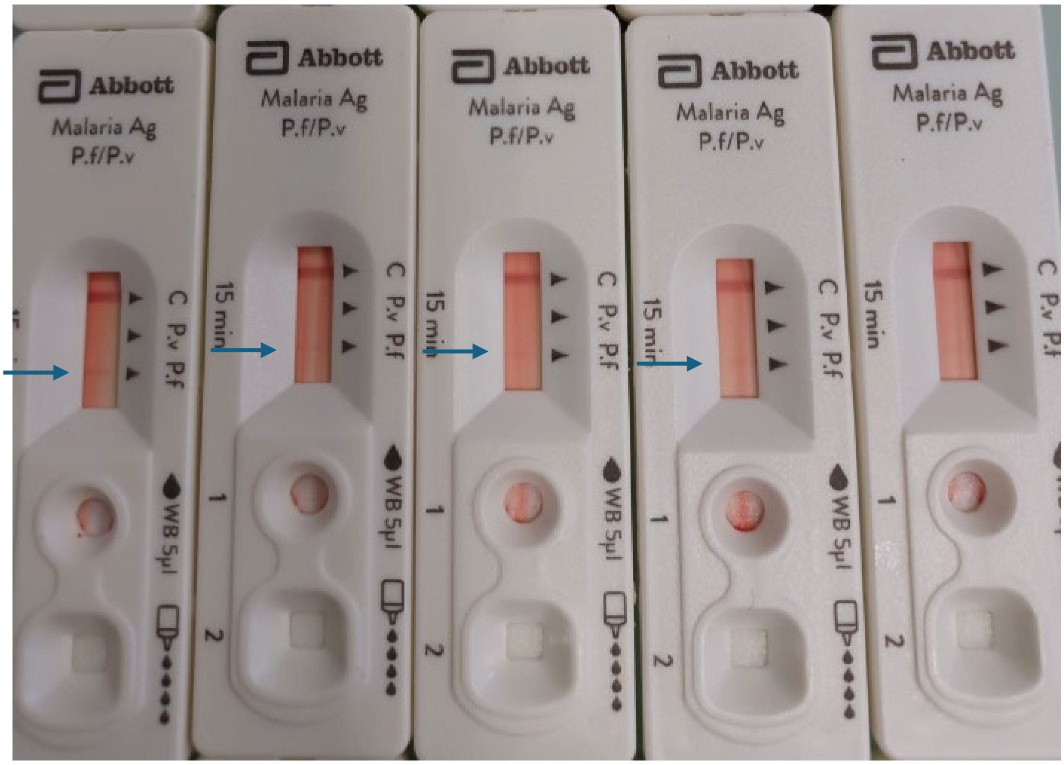

Lot 05DDI041AB (4/5 positive)

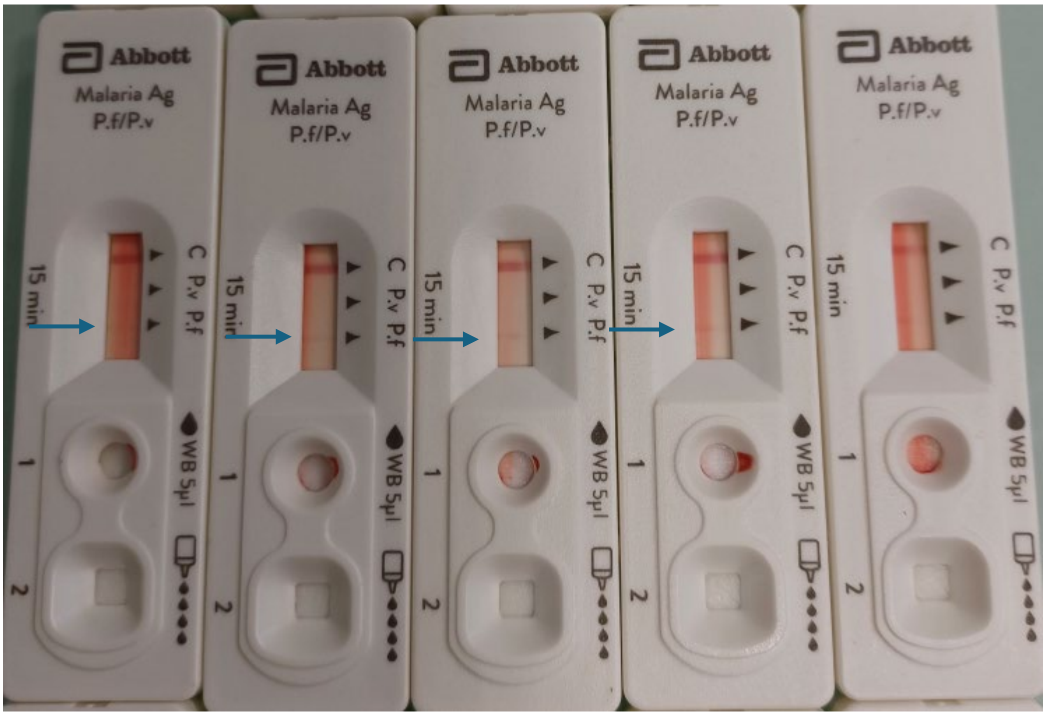

Lot 05DDI040AA (4/5 positive)

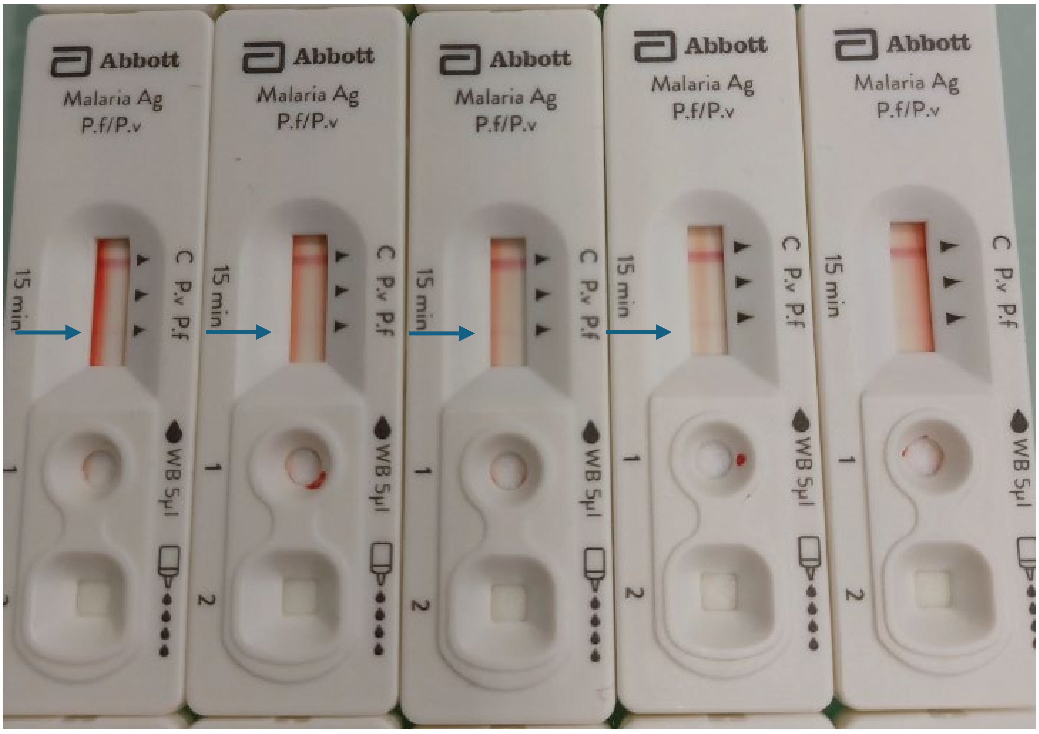

Lot 05DDI018BH (1/5 positive)

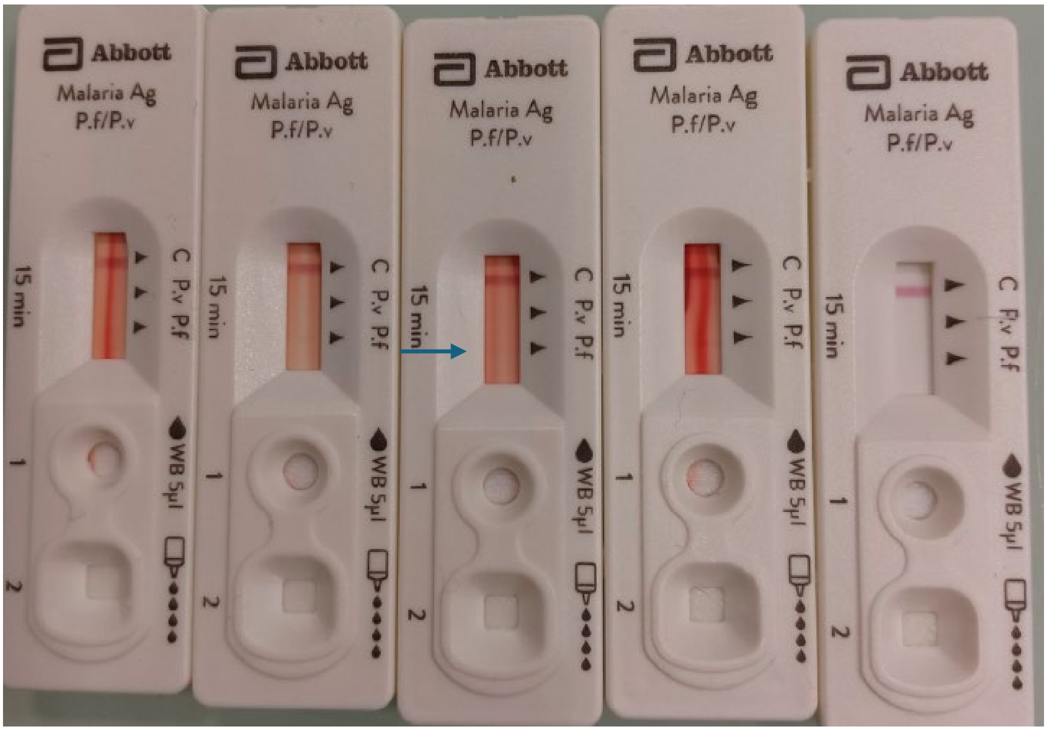

Figure S9. RDT results at 1,621 parasites/µL.
Improved detection across all lots with persistent inter-lot variability in line intensity (scores 1-2).

Lot 05DDI020BA (5/5 positive)

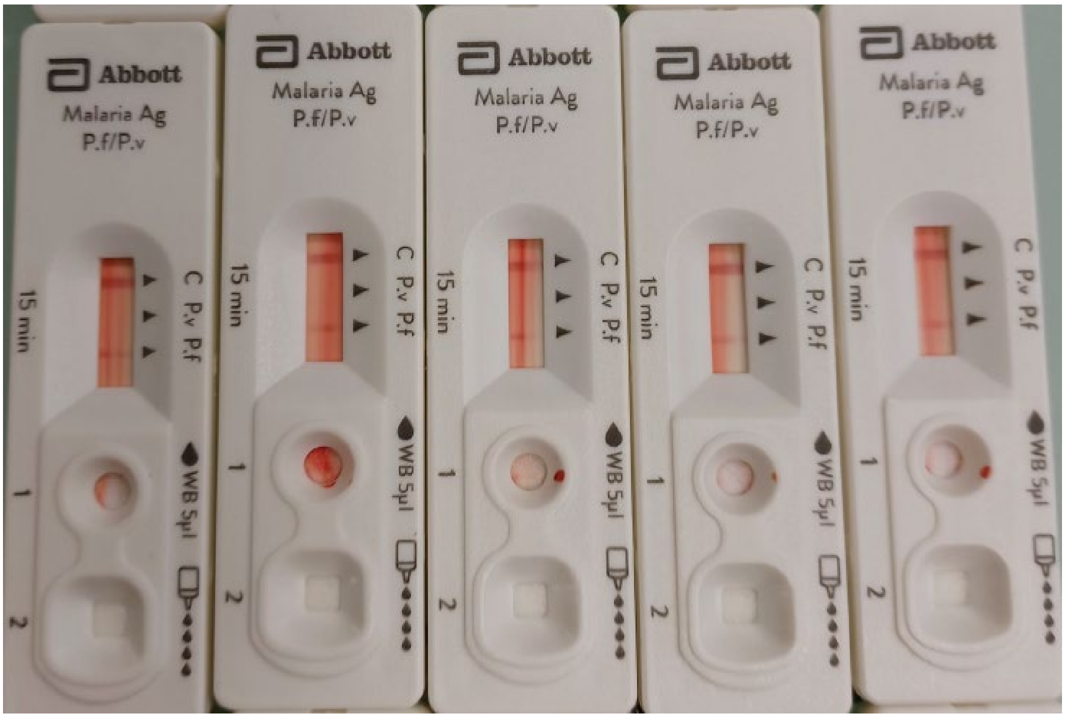

Lot 05DDI041AB (5/5 positive)

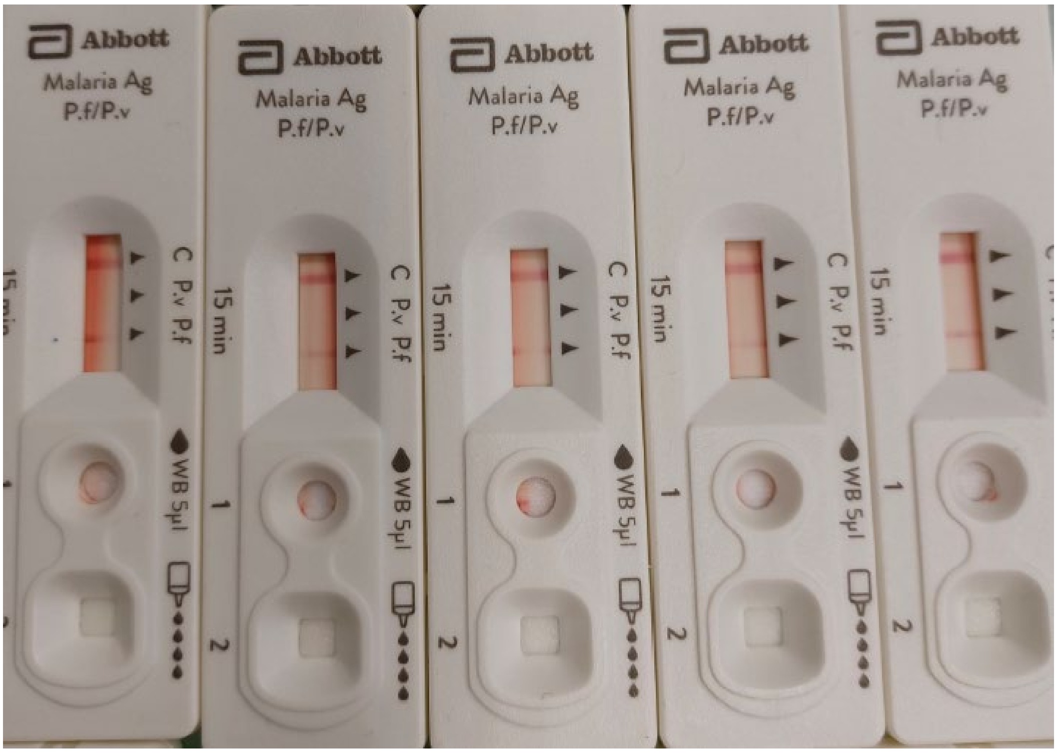

Lot 05DDI040AA (5/5 positive)

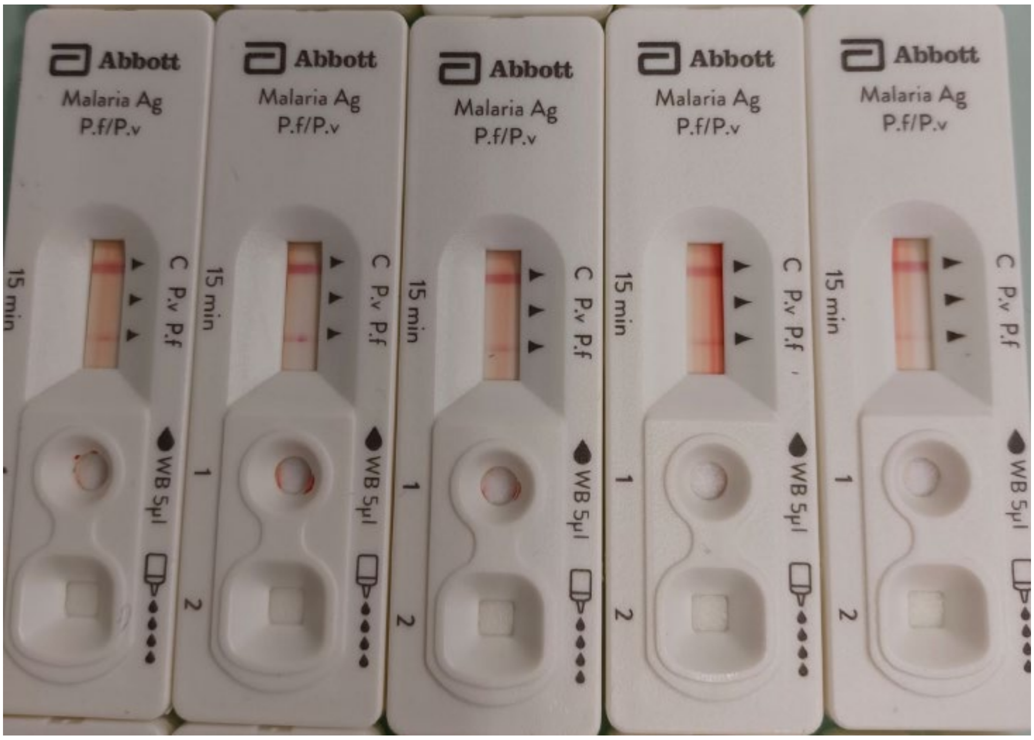

Lot 05DDI018BH (5/5 positive)

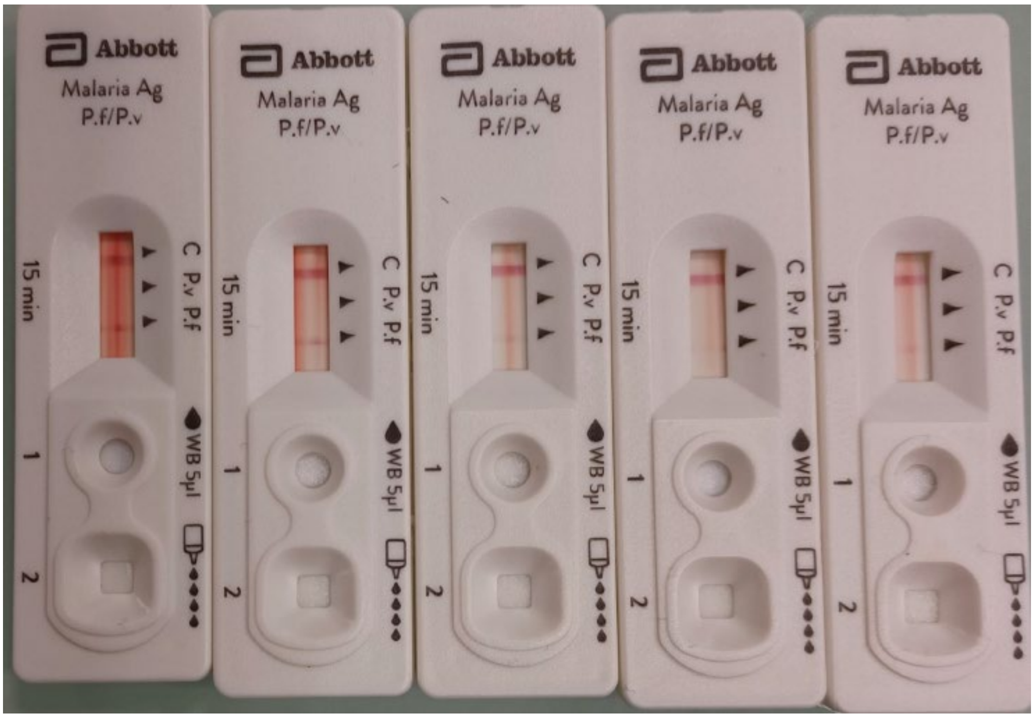

Figure S10. RDT results at 5,708 parasites/µL.
Consistent positive results across all lots (5/5 positive) with moderate line intensity (score 3).

Lot 05DDI020BA (5/5 positive)

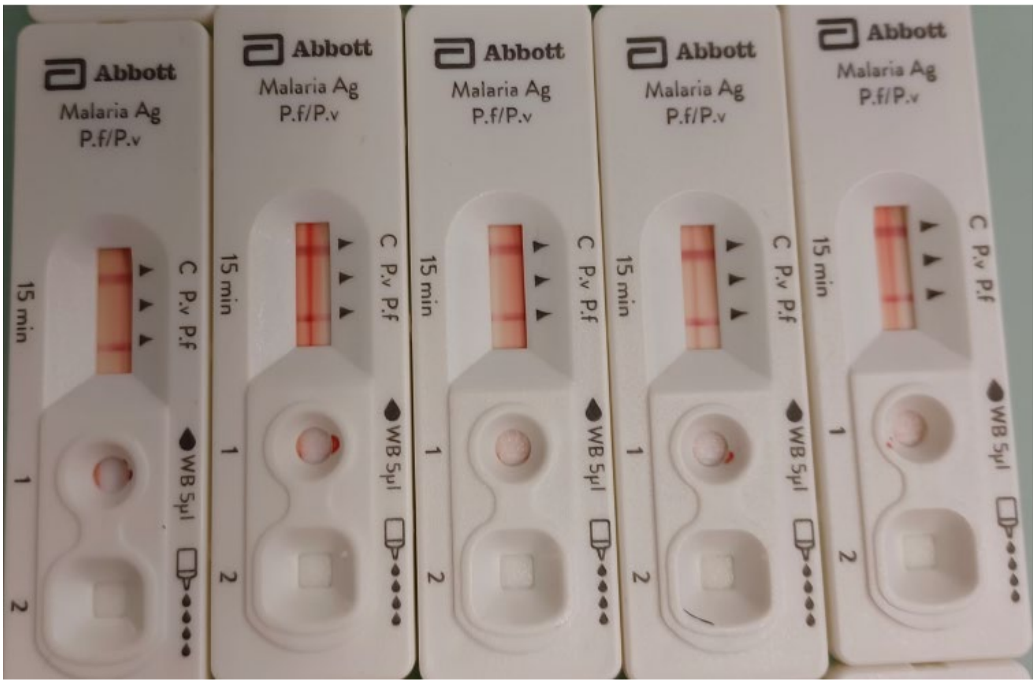

Lot 05DDI041AB (5/5 positive)

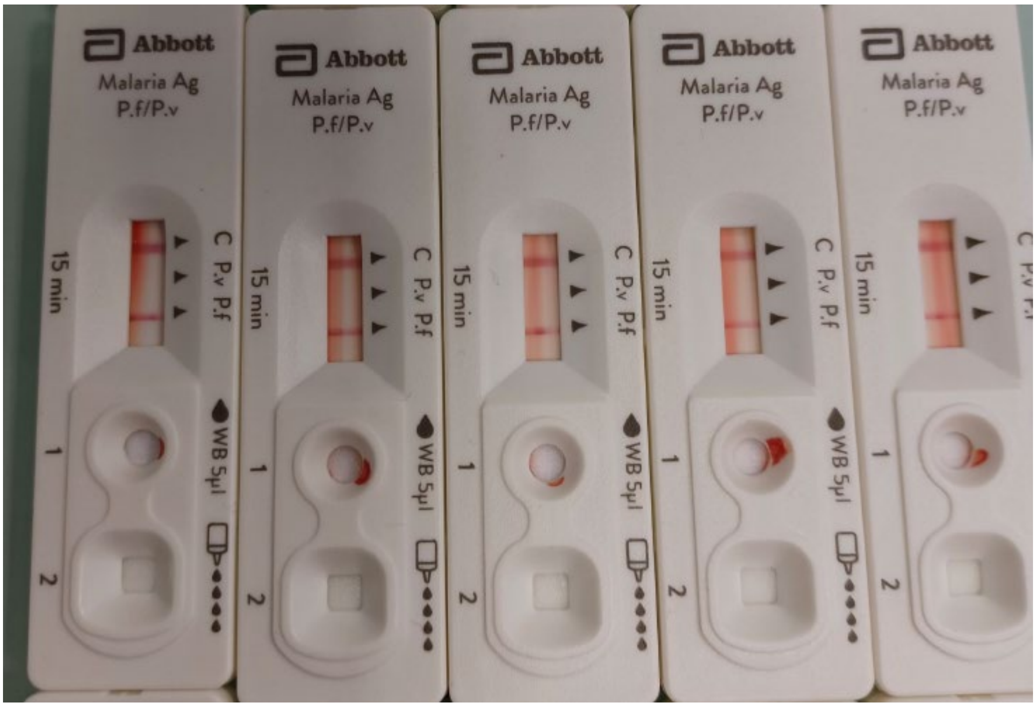

Lot 05DDI040AA (5/5 positive)

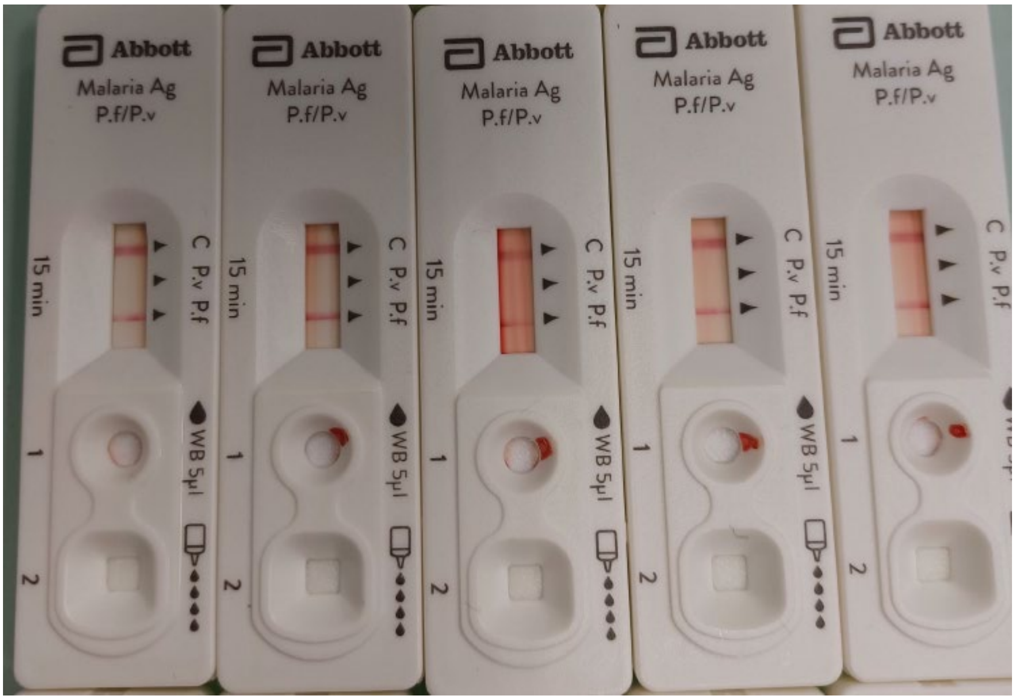

Lot 05DDI018BH (5/5 positive)

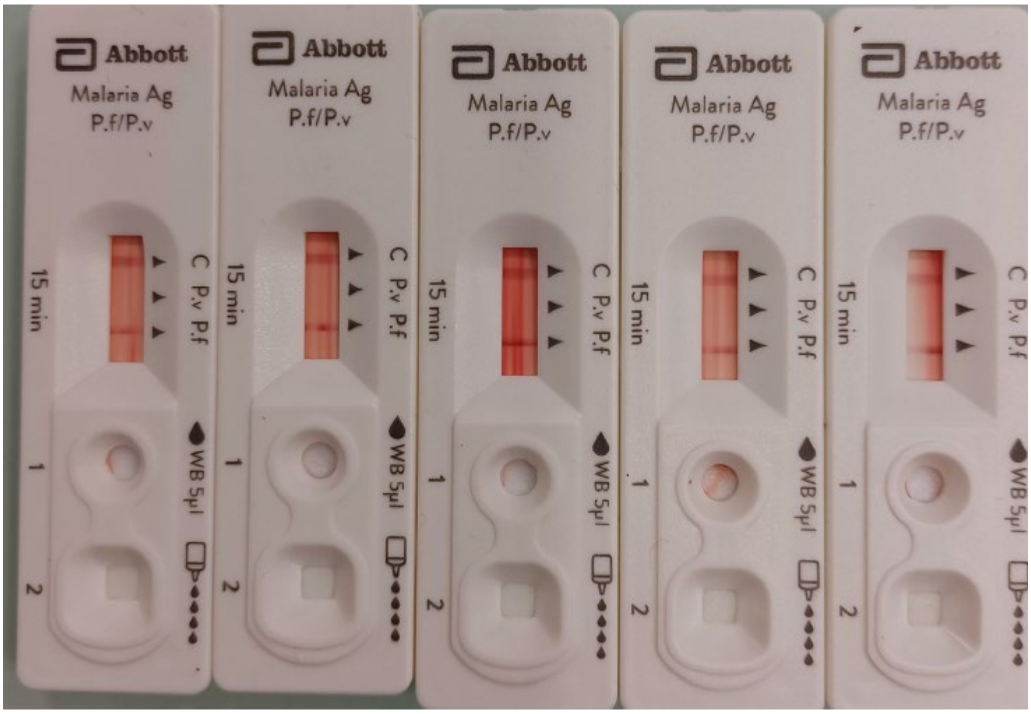

Figure S11. RDT results at 60,784 parasites/µL.
Maximum line intensity (score 4) across all lots and replicates.

Lot 05DDI020BA (5/5 positive)

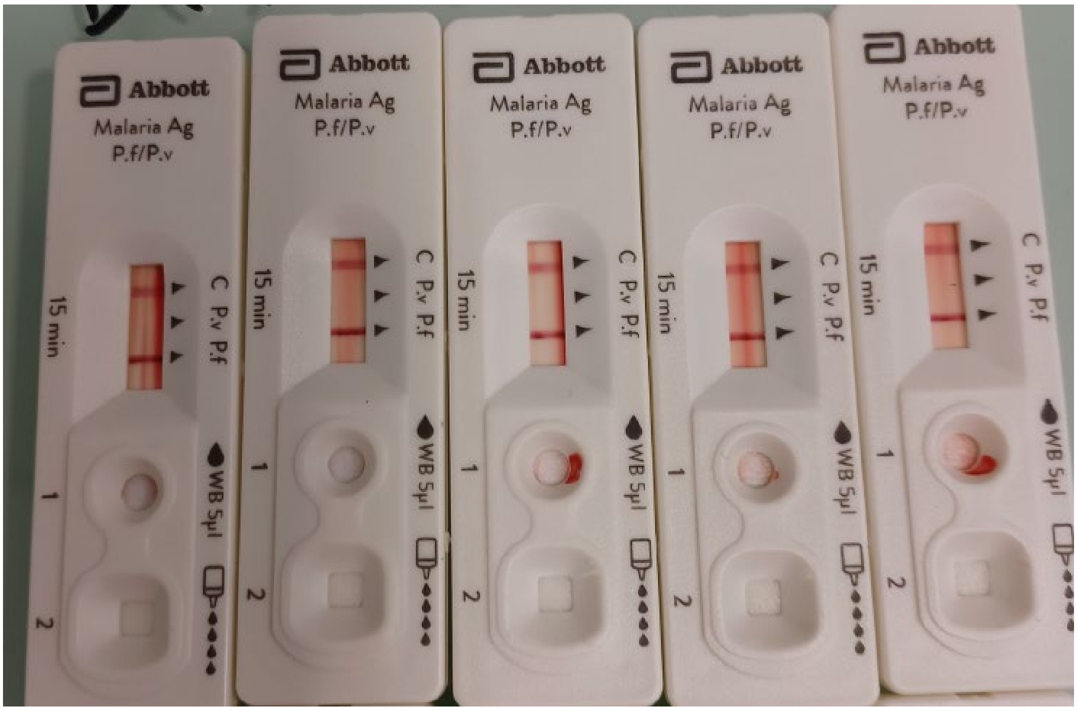

Lot 05DDI041AB (5/5 positive)

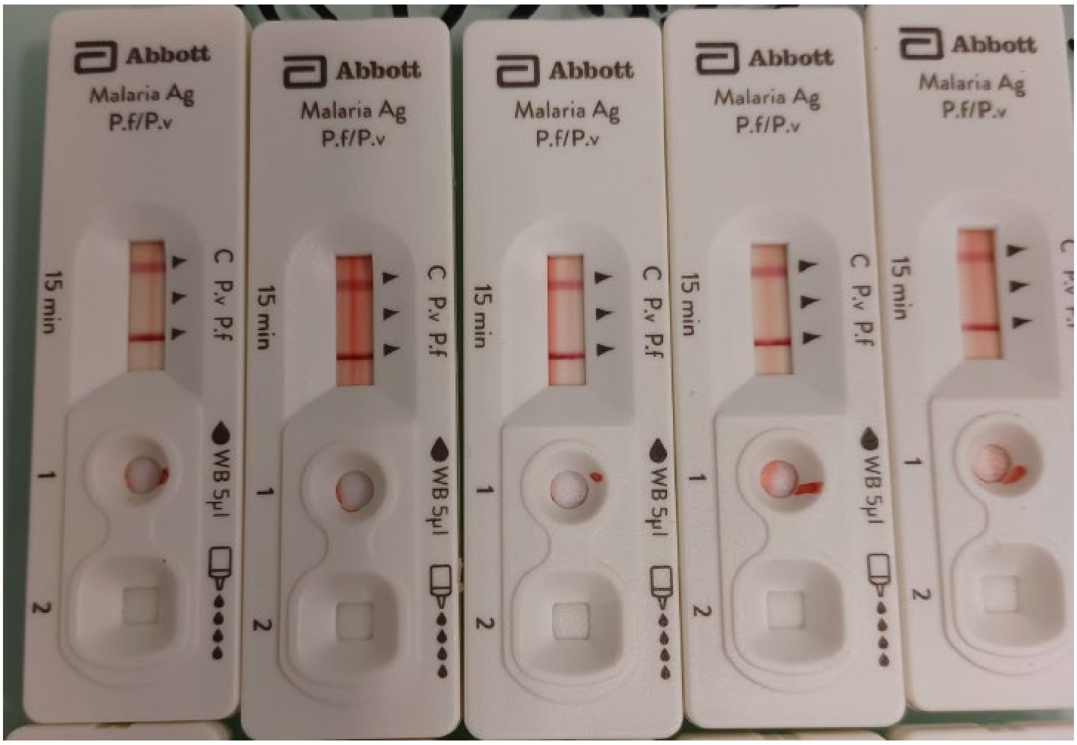

Lot 05DDI040AA (5/5 positive)

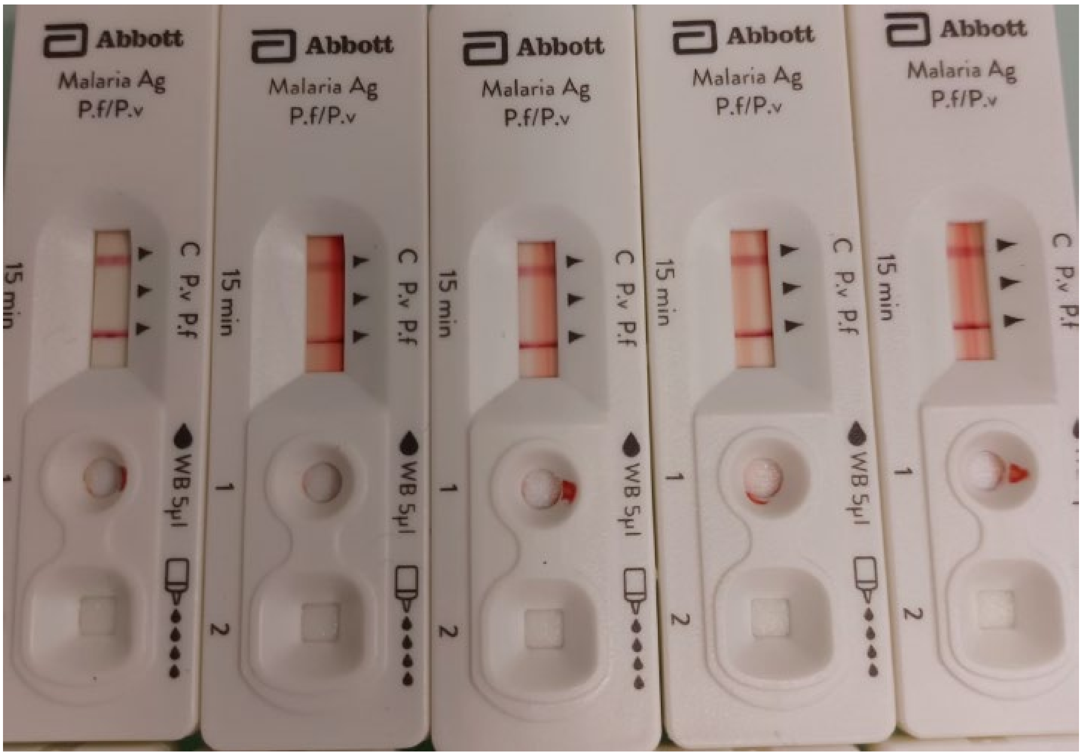

Lot 05DDI018BH (5/5 positive)

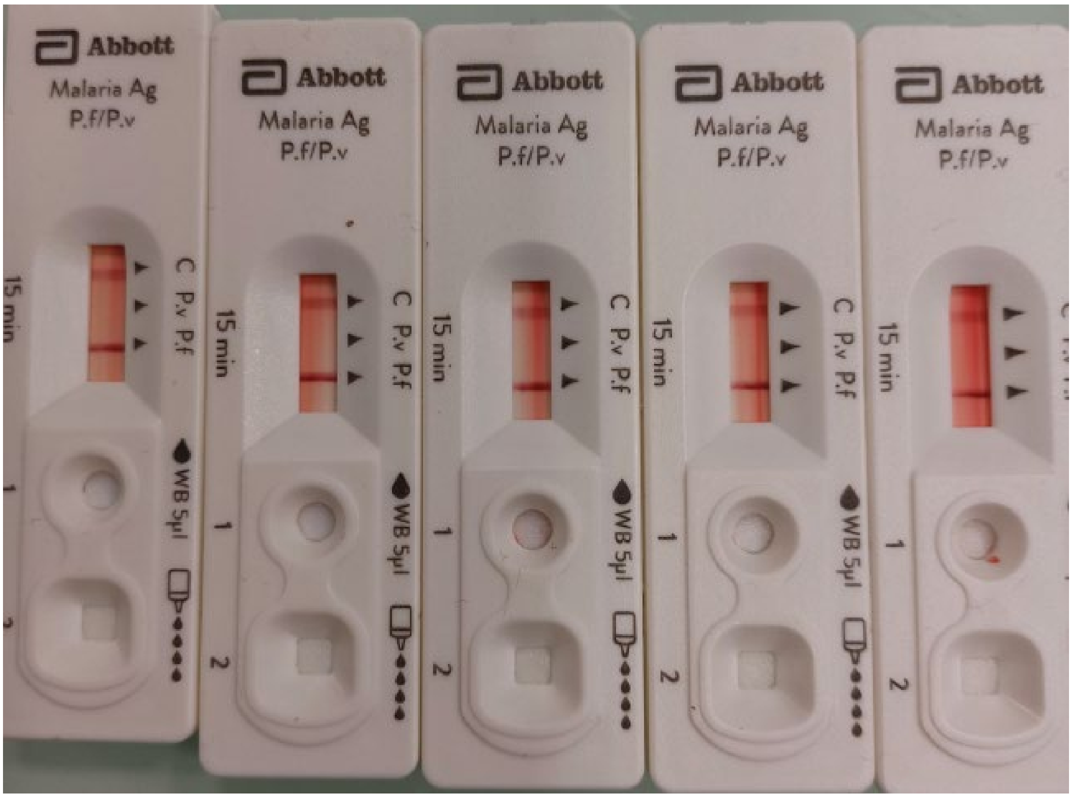

Migration defects.

Incomplete blood migration was frequently observed, where blood did not fully travel along the nitrocellulose strip, leaving a red background. In most cases, the background gradually cleared after 30 minutes, though this was not consistent across all RDTs.
